# Nutrient Composition, Physicobiochemical Analyses, Oxidative Stability and Antinutritional Assessment of Abundant Tropical Seaweeds from the Arabian Sea

**DOI:** 10.3390/plants12122302

**Published:** 2023-06-13

**Authors:** Babita Choudhary, Deepesh Khandwal, Nirmala Kumari Gupta, Jaykumar Patel, Avinash Mishra

**Affiliations:** 1 Division of Applied Phycology and Biotechnology, CSIR-Central Salt and Marine Chemicals Research Institute, G. B. Marg, Bhavnagar 364002, India; 2 Academy of Scientific and Innovative Research (AcSIR), Ghaziabad 201002, India

**Keywords:** antioxidants, bioactive compound, edible algae, minerals, oil oxidation, proximate composition, seaweeds

## Abstract

Foods enriched with nutritional compounds and biological activities, especially antioxidants, are considered healthier for human and/or animal consumption. Seaweeds are rich sources of biologically active metabolites and are used as functional foods. In this study, proximate compositions, physicobiochemical characteristics and oil oxidative stability were analyzed for 15 abundant tropical seaweeds (four green—*Acrosiphonia orientalis*, *Caulerpa scalpelliformis*, *Ulva fasciata*, *Ulva lactuca*; six brown—*Iyengaria stellata*, *Lobophora variegate*, *Padina boergesenii*, *Sargassum linearifolium*, *Spatoglossum asperum*, *Stoechospermum marginatum*; and five red—*Amphiroa anceps*, *Grateloupia indica*, *Halymenia porphyriformis*, *Scinaia carnosa*, *Solieria chordalis*). All seaweeds were analyzed for the proximate composition, including moisture content, ash content, total sugar content, total proteins, total lipids, crude fiber, carotenoid content, total chlorophyll content, proline, iodine content, nitrogen-free extract, total phenolic content and total flavonoid content. Green seaweeds showed higher nutritional proximate composition, followed by brown and red seaweeds. Among the different seaweeds, *Ulva*, *Caulerpa*, *Sargassum*, *Spatoglossum* and *Amphiroa* showed high nutritional proximate composition compared to other seaweeds. High cation scavenging, free radical scavenging and total reducing activities were observed for *Acrosiphonia*, *Caulerpa*, *Ulva*, *Sargassum*, *Spatoglossum* and *Iyengaria*. It was also observed that 15 tropical seaweeds contained negligible amounts of antinutritional compounds, including tannic acid, phytic acid, saponins, alkaloids and terpenoids. Nutritionally, green and brown seaweeds provided higher sources of energy (150–300 calories per 100 g) compared to red seaweeds (80–165 calories per 100 g). Additionally, this study also confirmed that tropical seaweeds improved the oxidative stability of food oils and, therefore, might be recommended as natural antioxidant additives. The overall results confirm that tropical seaweeds are potential sources of nutrition and antioxidants and may be explored as functional food, dietary supplementation or animal feed. Additionally, they may also be explored as food supplements for fortifying food products, as food toppings or for garnishing and seasoning foods. However, a human or animal toxicity analysis is required before any conclusive recommendation for daily food or feed intake can be made.

## 1. Introduction

Seaweeds have been traditional foods in many cultures since ancient periods, as well as being used as fertilizer, condiments and different phycocolloids, including agar, carrageenan and alginates [1]. The demand of consumers for high-quality healthy and nutritious foods and food products is continuously increasing. Currently, the fastest growing food production sector is aquaculture, which can help solve the problem of enhanced global food demand. Total aquaculture production increased globally to more than 120 million tons in the year 2019, and algae cultivation contributed about 30% [2]. Of the total global production of seaweeds of 35.8 million tons, about 1.1 million tons were obtained through wild collection, while 34.7 million tons were cultivated (farmed); this represented 97% of world seaweed production in 2019 [3]. Asia contributed 97.38% of world seaweed production in 2019, followed by the Americas (both North and South; 1.36%), Europe (0.8%), Africa (0.41%) and Oceania (0.05%) [2]. 

The world aquaculture economy amounted to USD 275 billion in 2019, and seaweeds accounted for 5.4% (USD 14.85 billion) [2]. According to global trade statistics, seaweeds produced USD 2.65 billion in foreign exchange in 2019 through the export of USD 1.74 billion of seaweed-based hydrocolloids and USD 0.91 billion of seaweeds [2]. Seaweeds have tremendous potential to meet the demand related to new technologies and have been used by food industries to produce novel functional foods [4,5]. Seaweeds are fit for human consumption due to their potential as a rich and sustainable resource of micro- and macronutrients for functional food, mostly in the regions where macroalgae (seaweeds) are a very important part of regular diets, such as in Japan, where seaweeds account for approximately one fifth of the population’s diets [6]. Seaweeds are considered a natural source of antioxidants and bioactive metabolites [7]. Seaweeds’ composition is still not well-known compared to land plants. Various factors affect their chemical and biological composition, including geographical localization, environmental conditions, maturity and species, which makes generalization very inadequate [8]. Seaweeds are also considered a valuable nutritional animal feed, especially as fish, oyster and poultry feed [9]. Previously, there were limited data on the digestibility and energy values of seaweeds for use in ruminants’ diets [10]. Today, seaweeds have become popular due to their potential suitability for feedstock production and use as animal feed additives [11].

More than 10,000 seaweed species have been reported globally and broadly classified into three taxonomic groups: green (more than 1800 macroalgal species under Chlorophyta); brown (about 2000 species under Ochrophyta/Phaeophyta); and red (approximately 7200 species under Rhodophyta). However, only 27 different seaweed species were cultivated in 2019 [2]. In worldwide seaweed cultivation in 2019, green, brown and red seaweeds accounted for 0.05%, 52.6% and 47.3%, respectively, in terms of production and 0.4%, 47.6% and 52%, respectively, in terms of value [2]. Seaweeds have mostly been cultivated or explored for use as human foods (soup and salads), food supplements, various food additives, edible hydrocolloids, animal feeds, biofertilizer, nutraceutical products, biodegradable packaging and cosmetics [12,13]. Most seaweed species do not contain any intrinsic toxins and are considered edible; about 700 seaweed species are documented as suitable or recommended for human consumption, including about 125 green, 195 brown and 345 red seaweeds [14].

Consumption of seaweeds in Western diets was previously limited to coastal communities and artisanal practices, but now it has gained wider consumer attention. The current surge of attention is fueled by awareness of the bioactive components of seaweed, which show possible benefits in nutraceutical applications [15] and as lucrative functional foods, with potential for the possible mitigation of metabolic risk factors, including hyperlipidemia, hypercholesterolemia and hyperglycemia [16]. The bioactive components significant for industrial applications include isolated proteins, polysaccharides, carotenoids, polyphenols and polyunsaturated fatty acids, minerals, proline, dietary fiber and vitamins [17]. Aside from these biologically active compounds, seaweeds are a natural and rich source of iodine, which is an essential micronutrient for human health and metabolism, such as in the production of thyroid hormones. Although iodine is essential, high consumption can cause problems for thyroids. Most iodine is acquired through the consumption of food that is either naturally rich or enriched with iodized salt [18]. Recent recommended daily intake (RDI) levels proposed by UNICEF, the WHO and the ICCIDD are based on health status, age and gender. Common iodine consumption guidelines are 90 µg per day for infants (first 12 months) and children 1–6 years old, 120 µg per day for children 7 to 12 years old and 200 µg per day for healthy adults (>12 years old) and pregnant or lactating woman [19,20].

Globally used edible oils contain high concentrations of unsaturated fatty acids, mainly polyunsaturated fatty acids, which are more susceptible to oxidation. Exposure of oils to high temperature and light can cause oxidation and amplify the peroxide value, causing the oils to become unpalatable [21]. Oil oxidation can produce unpleasant flavors, rancid odors and discoloration, as well as reducing the safety and nutritional value of degradation products, all of which affect human health. Lipid oxidation of oil can take place during either transportation or storage and can be stopped through the addition of antioxidants, which inhibit free radical generation and prevent rancidification. Currently, to prevent oxidative deterioration, the most common antioxidants are tert-butyl hydroquinone (TBHQ), butylated hydroxytoluene (BHT) and butylated hydroxyanisole (BHA). These are globally used as food additives. Recent studies have shown that these synthetic antioxidants may be implicated in various health risks that can affect the spleen, liver and lungs [22]. To overcome these problems, researchers have turned to natural sources of antioxidants to replace these synthetic antioxidants [23].

Previously, we demonstrated the antioxidant and nutraceutical potential of abundantly available tropical seaweeds from the Saurashtra coast of the Arabian Sea and their application as dietary supplements or functional foods [24,25,26]. Among the abundant tropical seaweeds studied, *Acrosiphonia* spp., *Caulerpa* spp., *Grateloupia* spp., *Padina* spp., *Sargassum* spp., *Spatoglossum* spp. and *Ulva* spp. are considerd edible seaweeds [14,27]. There are several reports on different seaweed compounds, including amino acids, fatty acids, phenolic compounds, flavonoids and other bioactive molecules, but there are no reports so far on the nutritional composition and physicobiochemical characteristics of the abundant tropical seaweeds along the Arabian Sea coast. Furthermore, there is a lack of knowledge about the nutritional and antinutritional composition of the abundant tropical wild seaweeds. Therefore, with the aim of nutritional profiling, the present study was performed to determine the proximate composition, different nutritional ingredients, physicobiochemical activities, oxidative stability and antinutritional contents of 15 abundant wild seaweeds. As stated, there is no comprehensive information available on the nutritional composition so far; our results revealed the nutritional and antioxidant potential of tropical wild seaweeds, which need to be explored further for use as animal feed, dietary supplements, food ingredients or functional food.

## 2. Materials and Methods

### 2.1. Sample Collection

Fifteen abundantly available wild tropical seaweeds (four green—*Acrosiphonia orientalis* (J.Agardh) P.C.Silva (ACR), *Caulerpa scalpelliformis* f. *dwarkensis* Børgesen (CAU), *Ulva fasciata* Delile (UF) and *Ulva lactuca* Linnaeus (UL); six brown—*Iyengaria stellata* (Børgesen) Børgesen (IYN), *Lobophora variegata* (J.V.Lamouroux) Womersley ex E.C. Oliveira (LOB), *Padina boergesenii* Allender & Kraft (PAD), *Sargassum linearifolium* (Turner) C. Agardh (SAR), *Spatoglossum asperum* J. Agardh (SPA) and *Stoechospermum marginatum* (J.V. Lamouroux) J. Agardh (STO); and five red—*Amphiroa anceps* (Lamarck) Decaisne (AMP), *Grateloupia indica* Børgesen (GRA), *Halymenia porphyriformis* P.G. Parkinson (HAL), *Scinaia carnosa* (Kützing) J. Agardh (SCI) and *Solieria chordalis* (C. Agardh) J. Agardh (SOL)) were collected from their natural habitat (Supplementary Table S1; Saurashtra coast of the Arabian Sea, Gujarat, India) [25,28] in the months of November and December 2021, identified [29], transported to a laboratory under controlled conditions [24], processed [25] and stored (at −80 °C) until further use.

### 2.2. Proximate Composition Analysis 

#### 2.2.1. Determination of Moisture Content, Ash Content, Total Proteins and Total Carbohydrates

The moisture content of the fresh material was determined by oven-drying the seaweeds at 60 °C until constant weight was achieved and subtracting the dry weight values from the wet weight values. Seaweed ash content was estimated gravimetrically after heating samples at 550 °C in a muffle furnace for 24 h. Total proteins were determined, and protein content was quantified using the Bradford assay. Total carbohydrates and uronic acid were determined using a spectrophotometer (absorbance at 490 for carbohydrates and 480 for uronic acid), following the phenol sulfuric acid method [30,31].

#### 2.2.2. Determination of Crude Fiber Content

To determine crude fiber content [32], seaweed samples (500 mg; *W*0) were digested with 150 mL sulfuric acid (0.128 M) at 100 °C for 30 min. Digested mixtures were filtered and washed twice using 25 mL hot distilled water. Washed samples were further treated with 150 mL potassium hydroxide (0.223 M), filtered, washed twice with 25 mL hot distilled water and dried. Finally, samples were washed with acetone and incubated for 2 h in an oven at 100 °C. Samples were cooled and weighed (*W*1). The residue was ashed at 550 °C for 3 h in a muffle furnace. The ashed residue was desiccated, cooled and weighed (*W*2). Crude fiber was calculated in percent using the following equation [32]:

$$Percent\;crude\;fibre=\left[\frac{W1-W2}{W0}\right]\times 100$$

#### 2.2.3. Determination of Total Lipids

Total lipid content was estimated using the chloroform–methanol method [33,34]. About 10 mg of seaweed sample was mixed with a mixture of 5 mL chloroform–methanol (2:1, *v*/*v*) and incubated at room temperature for 24 h in the dark. The incubated mixture was filtered, and the crude extract was washed with 20% of its volume in 0.9% NaCl solution and vigorously mixed for phase separation. The lower phase containing the total lipids was transferred to a pre-weighed beaker (*W_empty_*), which was kept on a hot plate until complete removal of the solution; finally, the weight of the beaker with the residue (*W_residue_*) was estimated. Total lipid content was estimated by subtracting with the weight of the empty beaker (*W_empty_*) from the weight of the beaker containing the residue (*W_residue_*) [33,34].

#### 2.2.4. Estimation of Nitrogen-Free Extract

The nitrogen-free extract (NFE) of the seaweed was calculated based on the percent weight difference of crude fiber, crude protein, crude ash and crude lipid, as per the following equation [35]:

$$NFE\left(\%\right)=100-(\%\;Crude\;fibre+\%\;Crude\;protein+\%\;Crude\;ash+\%\;Crude\;lipid)$$

#### 2.2.5. Determination of Proline Content

Proline content was determined based on the method described by Bates et al. [36] using ninhydrin reagent. About 500 mg of seaweed sample was mixed with 10 mL sulfosalicyclic acid and filtered. Equal amounts of filtrate, ninhydrin and glacial acetic acid were added, and the mixture was incubated for 60 min at 100 °C. The incubated mixture was cooled and subjected to extraction with toluene via vigorous mixing. The colored solution was collected from the aqueous phase and kept at room temperature for 5 min. Absorbance was measured at 520 nm and proline content was determined using a standard curve produced with a known concentration of proline [36].

#### 2.2.6. Determination of Chlorophyll Content

Total chlorophyll and carotenoid contents were determined based on the methods described by Inskeep and Bloom [37] and Chamovitz et al. [38], respectively. About 20 mg of seaweed sample was mixed with 1 mL of N,N-dimethyl formamide and the mixture was kept at 4 °C in the dark for 30 min with gentle shaking. The sample was centrifuged for 15 min at 9000× *g* and 4 °C, and the supernatant was collected. Absorbance (Abs) was recorded at 664.5, 664, 647 and 461 nm, and total chlorophyll and carotenoid contents were determined using the following equations:

$$Total\;chlorophyll\;content\;(mg/L)=17.90A_{647}+8.08A_{664.5}$$

$$Total\;Carotinoid\;content\;\left(mg/L\right)=[A_{461}-(0.046A_{664})]\;\times \;4$$

#### 2.2.7. Total Phenolic Content

Total phenolic content was determined based on the method described by Singleton and Rossi [39] and optimized by Tanna et al. [25]. To determine the total phenolic content (TPC), 100 mg of seaweed sample was subjected to extraction with a mixture of 10 mL acetone and hydrochloric acid (80% and 1%, respectively) for 6 h in an orbital shaker with continuous shaking at 200 revolutions per minute (rpm). An aliquot of 40 µL of this mixture was placed in another tube and mixed with 360 µL of deionized water; further, 200 µL of 2 M Folin–Ciocâlteu reagent and 20% Na_2_CO_3_ solution (600 µL) were added. The solution was mixed thoroughly and incubated at 40 °C for 30 min in a dark place. Incubated samples were centrifuged at 9000× *g* for 2 min at room temperature. The supernatant was used to measure the total phenolic content by recording the absorbance at 765 nm [25]. TPC was determined against a standard curve of gallic acid and expressed as milligrams gallic acid equivalent per gram dry weight (mg GAE g^−1^ Dw) of seaweed sample [24].

#### 2.2.8. Total Flavonoid Content

Total flavonoid content (TFC) was determined by following the colorimetric method [40]. About 100 mg of seaweed sample was mixed with 20 µL aluminum chloride (10%), 20 µL potassium acetate (1 M) and 180 µL distilled water (all freshly prepared) and incubated at room temperature for 30 min. The absorbance was measured at 415 nm. TFC was calculated using a calibration curve for quercetin solution prepared at different concentrations. TFC was expressed as milligrams quercetin equivalent per gram dry weight (mg quercetin g^−1^ Dw) of seaweed sample [26].

#### 2.2.9. Determination of Mineral Content

Inductively coupled plasma mass spectrometry analysis was performed to determine the mineral composition [41]. About 1 g of dry seaweed sample was ignited and incinerated in a muffle furnace over 24 h at 550 °C to remove the carbon. The ash was dissolved with 5 mL of aqua regia and hydrogen peroxide (HNO_3_:HCl:H_2_O_2_; 1:2:3) solution. The mixture was vortexed, filtered and subjected to acid digestion on a hot plate until the brown fumes gently disappeared. Distilled water was added to the acid-digested mixture until it became colorless. The sample was filtered and the volume made up with deionized water, and then it was subjected to inductive coupled plasma mass spectrometry analysis [41].

#### 2.2.10. Estimation of Iodine Content

The ion chromatography (IC) method was used to estimate iodine content [42,43]. To determine the iodine content, about 200 mg of seaweed sample was digested in 17 M KOH at 600 °C for 4 h. The resultant ash was reconstituted in 2.5 mL of deionized water and diluted with 2.5 mL of methanol [43]. To measure the total iodine content, the sample was subjected to ion chromatography [42]. For the IC of the iodine content, a Thermo Fisher ICS 5000 instrument was used with an AS 11 HC column and amperometry detector. The flow rate was 1 mL min^−1^ and the eluent was 20 mM NaOH.

### 2.3. Physicochemical Analyses

#### 2.3.1. Determination of Water-Swelling Capacity

The seaweed sample (200 mg dried power) was vigorously mixed with 20 mL distilled water. The volume was measured (before swelling), and it was kept for 24 h at room temperature (25 °C) [44]. The sample volume was again measured, and the water-swelling capacity (WSC) was calculated and expressed as mL of swollen sample occupied per gram dry weight of the sample using the following equation:

$$WSC\;(mL\;g^{-1})=\frac{{Vol}_{after\;swelling}-{Vol}_{before\;swelling}}{W_{sample}}$$

#### 2.3.2. Estimation of Water-Holding Capacity

To determine the water-holding capacity (WHC), the dry seaweed sample (200 mg dried power) was vigorously mixed with 20 mL of distilled water in pre-weighed tubes and kept for 24 h at room temperature (25 °C) with continuous shaking. The mixture was centrifuged at 3000× *g* for 25 min at room temperature, and the residue was collected and weighed. The WHC was calculated using the following equation and expressed as grams of water held per gram dry weight of the sample [45]:

$$WHC\;({g\;g}^{-1})=\frac{Wet\;weight-Dry\;weight}{Dry\;weight}$$

#### 2.3.3. Determination of Oil-Holding Capacity

Corn oil (10 g) was mixed with 3 g of sample in a pre-weighed centrifugation tube to determine the oil-holding capacity of the seaweed samples [46]. The mixture was vortexed and the tubes were left at room temperature for 30 min with constant agitation. The mixture was centrifuged at 2500× *g* for 30 min at room temperature. The oil supernatant was removed, and the residue was weighed. Oil-holding capacity (OHC) was expressed as grams of oil held per gram dry weight of the sample [46]. The density of oil is 0.92 g mL^−1^.

$$OHC\;({gg}^{-1})=\frac{Wet\;weight\;residue-Dry\;weight\;of\;sample}{Dry\;weight}$$

### 2.4. Estimation of Oxidation Stability of Oil under Accelerated Conditions

To determine the effectiveness of seaweed against lipid oxidation, the oxidative stability of oil under accelerated conditions was analyzed [21]. The seaweed sample was mixed with 1.5 mL ethanol (96% *v*/*v*) to ensure appropriate dispersion in oil. Simultaneously, a set of experiments were performed with the widely used synthetic antioxidant BHT instead of seaweed samples. The mixture was incubated in ultrasonic water bath for 10 min and then subjected to evaporation under a vacuum. The sample or the BHT was added to crude peanut oil at a concentration of 500 ppm. Refined oil, without the addition of any antioxidant, was used as a control. The oil-mix samples (control, with 500 ppm BHT or with 500 ppm seaweed) were kept at 60 °C with continuous shaking (100 rpm) for different time ranges (0–16 days). Lipid oxidation was analyzed by measuring different indicators (chemical indices), including peroxide value, p-anisidine value, thiobarbituric acid-reactive substance (TBARS) value and conjugated dienes, as described in Section 2.4.1, Section 2.4.2, Section 2.4.3, Section 2.4.4, Section 2.4.5. The total oxidation (TOTOX) was calculated for each oil-mix sample.

#### 2.4.1. Determination of Peroxide Value

For the measurement of the peroxide value (PV), 500 mg of the oil-mix sample was added to 10 mL of trichloromethane, followed by the addition of 15 mL acetic acid and 1 mL saturated aqueous solution of potassium iodide. Samples were slightly agitated for 1 min and kept in the dark for 5 min. About 75 mL distilled water was added and vigorously mixed. The mixture was titrated against sodium thiosulfate (0.01 N) until the yellow color disappeared [21]. The control was also analyzed under similar conditions. The peroxide value was expressed as milli-equivalents O_2_ kg^−1^ (mEq O_2_ kg^−1^) and calculated using the following formula:

$$PV=\frac{V\times N\times 1000}{W}$$

where *V* is the volume (in mL) of sodium thiosulfate consumed in the titration, *N* (0.01) is the normality of the sodium thiosulfate solution and *W* is the weight (0.5 g) of the sample.

#### 2.4.2. Determination of p-Anisidine Value

The p-anisidine value (*AV*) was determined according to an IUPAC-approved method [23]. About 1 g of oil-mix sample was dissolved in 25 mL of isooctane. The solution was allowed to react with p-anisidine solution (1 mL) in acetic acid (0.25%, *w*/*v*) for 10 min to produce a colored complex [23]. Absorbance was read at 350 nm and the *AV* was calculated as follows:

$$AV=25\times \left[\frac{1.2\times \left(E_{b}-E_{a}\right)}{W}\right]$$

where *E_b_* is the absorbance of the oil solution (before reaction), *E_a_* is the absorbance of the oil–anisidine reaction solution and *W* is the weight of the sample (in grams).

#### 2.4.3. Determination of Conjugated Dienes 

To determine the conjugated dienes (CDs) value, the oil-mix sample was diluted with hexane in a 25 mL volumetric flask. The absorbance of the diluted sample was measured at 233 nm, and the CDs value, expressed as a percentage of the conjugated dienoic acid, was calculated as follows [21]:

$$CD=\frac{0.84\times {Abs}_{233}}{b\times c-K_{0}}$$

where *Abs* is the absorbance observed at 233 nm, *b* is the cell length of the cuvette in cm, *c* is the concentration of the diluted sample (g L^−1^) and *K*_0_ is the absorptivity in relation to acid or ester groups. In this case, *b*, *c* and *K*_0_ were 12.776 cm, 0.5 g L^–1^ and 0.03, respectively.

#### 2.4.4. Determination of Total Oxidation Value

The total oxidation (*TOTOX*) value was determined with the following equation:

TOTOX=AV+2PV

#### 2.4.5. Determination of Thiobarbituricacid-Reactive Substance Value

For thiobarbituric acid-reactive substance (*TBARS*) value estimation, 200 mg of the sample was mixed with 25 mL 1-butanol in a volumetric flask. Then, 5 mL was transferred to another tube, and 5 mL freshly prepared TBA reagent (200 mg TBA in 100 mL butanol) was added and heated at 95 °C in a water bath for 120 min. A pink color developed, the mixture was allowed to cool at room temperature and the absorbance was measured at 532 nm [21]. *TBARS* values were calculated with the equation given below:

$$TBARS=\frac{{Abs}_{532}\times 0.415}{W_{sample}}$$

### 2.5. Biological Activity

The total antioxidant, scavenging and reducing activities of the seaweeds were determined using previously optimized methods [47,48,49]. Total antioxidant and scavenging activities were measured by inhibition of 2,2′-azino-bis(3-ethylbenzothiazoline-6-sulphonic acid) (ABTS) and 2,2-diphenyl-1-picrylhydrazyl (DPPH) free radicals at 734 and 514 nm, respectively, and 6-hydroxy-2,5,7,8-tetramethylchroman-2-carboxylic acid (Trolox) was used as standard. 

#### 2.5.1. Total Antioxidant Activity 

For total antioxidant activity, ABTS free radicals were generated by incubating ABTS diammonium salt (7 mM) with potassium persulfate (2.45 mM) for 12–16 h in the dark [50]. These ABTS free radicals were diluted with water until an absorbance of 0.70 ± 0.02 at 734 nm was obtained. Different amounts of seaweeds (1–5 mg) or Trolox (used as standard) were mixed with ABTS free radicals (1 mL). The samples were incubated for 90 min at room temperature, and absorbance was read at 734 nm. Total antioxidant activity was calculated by comparing the absorbance of samples with the standard and percentage inhibition was calculated [50].

#### 2.5.2. Scavenging Activity 

For scavenging activity, DPPH solution was diluted with methanol until an absorbance 0.98 ± 0.02 at 517 nm was obtained [50]. Different amounts of seaweeds (1–5 mg) or Trolox (used as standard) were mixed with DPPH (1 mL). The samples were incubated overnight at room temperature in the dark, and absorbance was read at 517 nm. Scavenging activity was measured by comparing the absorbance of samples with the standard and percentage inhibition was calculated [50].

#### 2.5.3. Reducing Activity 

The reducing power of the seaweed sample was determined according to their capacity to reduce ferric ions [51]. FeSO_4_ was used as a standard, and absorbance was read at 593 nm. The ferric reducing antioxidant power (FRAP) assay reagent was prepared from 10 mmol L^−1^ tripyridyltriazine (TPTZ), 40 mmol L^−1^ hydrochloric acid, 20 mmol L^−1^ ferric chloride and 0.3 mol L^−1^ acetate buffer (pH 3.6). Different amounts of seaweeds (1–5 mg) or FeSO_4_ (used as standard) were mixed with a FRAP assay reagent (1 mL) and incubated for 10 min at 37 °C. Absorbance was measured at 593 nm, and total reducing capacity was determined over the linear range of the standard curve [51]. 

The half-maximal effective concentration (EC_50_) was calculated via linear regression graph analysis of the dose–response curve for each bioactivity.

### 2.6. Antinutritional Activities

#### 2.6.1. Tannin Estimation

Tannin was estimated based on the method described by Saxena et al. [52]. Powdered seaweed (0.5 g) was transferred to a conical flask and 750 mL of distilled water was added. Samples were incubated for 3 min at boiling temperature and centrifuged at 2000× *g* for 20 min. Supernatant was collected and the volume was topped up to 1000 mL. The extracted sample (1 mL) was further diluted by adding 75 mL distilled water. Folin–Denis reagent (5 mL) and sodium carbonate (10 mL) were added to the diluted sample and the volume was topped up to 100 mL using distilled water. The sample was incubated for 30 min and absorbance was recorded at 700 nm. Tannic acid was used as a standard (10–100 µg). The tannin content (µg g^−1^) of the sample was calculated with a regression graph for the tannic acid solution [52].

#### 2.6.2. Phytic Acid Determination

Phytic acid was estimated based on the method described by Gao et al. [53]. First, 10 mL of HCl (2.4%; 0.64 N) was added to the seaweed samples (0.5 g), and they were vigorously mixed and then incubated at 300 rpm for 16 h on a platform shaker at room temperature. Incubated samples were centrifuged for 20 min at 10 °C and 3000× *g* and the supernatant was collected by filtering through Whatman filter paper into a tube that contained pre-weighed NaCl (1 g). The solution was vigorously mixed to dissolve the salt and kept on a shaker platform for 20 min at 300 rpm. The sample was kept idle to allow it to settle for 60 min at 4 °C. The sample was centrifuged at 10 °C and 3000× *g* for 20 min, the supernatant was collected and 1 mL of this supernatant was diluted with deionized water to 25 mL. The diluted sample (3 mL) was mixed with the Wade reagent (1 mL), vortexed thoroughly and centrifuged for 10 min at 10 °C (3000× *g*). Absorbance was recorded at 500 nm and phytic acid content was calculated with the regression curve for the standard [53].

#### 2.6.3. Determination of Saponins

To determine the saponins, 100 mg of powdered seaweed was mixed with distilled water (20 mL) and incubated in boiling water bath for 5 min. The sample was cooled to room temperature and filtered through a nylon membrane, and the volume was brought up to 100 mL with distilled water. Various dilutions ranging from 10^−1^ to 10^−5^ were produced from this filtered solution, vigorously mixed and incubated at room temperature for 15 min. Saponin was determined from the presence of persistent foam [54].

#### 2.6.4. Determination of Alkaloids

Seaweed samples (1 g) were treated with 1% (*v*/*v*) H_2_SO_4_ (10 mL) followed by incubation in a boiling water bath for 2 min. The mixture was filtered with Whatman paper and 1 mL of filtrate was mixed with 40 mL of Dragendorff reagent. The presence of alkaloids was determined from the appearance of an orange-red precipitate [54].

#### 2.6.5. Determination of Terpenoids

Terpenoids were extracted from seaweeds by adding 1 g of sample to the 10 mL of methanol followed by proper mixing. Further chloroform (2 mL) and concentrated H_2_SO_4_ (3 mL) were added to the extract (5 mL). The presence of terpenoids in seaweed samples was confirmed by the appearance of a reddish-brown color [54].

### 2.7. Statistical Analysis

Data are shown as means ± SE. ANOVA and Tukey’s honest significant difference (HSD) were applied for statistical analysis. Statistical differences are expressed with different letters at a significance *p* < 0.05.

## 3. Results

### 3.1. Proximate Composition

Proximate composition analyses included estimation of moisture content, ash content, total sugar content, total protein content, total lipids, crude fiber, carotenoid content, total chlorophyll content, proline, iodine content, nitrogen-free extract, total phenolic content and total flavonoid content (Table 1). Among the tropical seaweeds, the red seaweed AMP showed the lowest moisture content (9 ± 1%), while most seaweeds contained moisture contents in the range of 15–25% and the maximum moisture content of 27 ± 1% was observed in the green seaweed UF after drying. Interestingly, the highest ash content of 75 ± 2% was detected in AMP, followed by IYN (51 ± 2%). The lower range of ash content (15–25%) was observed in green seaweeds, followed by brown (25–32%; except IYN), and a higher range of ash content > 40% (except GRA (39 ± 2%) and HAL (17 ± 1%)) was detected in red seaweeds. 

Among all 15 tropical seaweeds, the 3 seaweeds UL (56 ± 2%), SAR (53 ± 3%) and SPA (49 ± 0.5%) contained the highest total sugar content, while IYN (9 ± 1%) and AMP (9 ± 1%) contained the lowest total sugar content. Most seaweeds contained total sugars in the range of 15–30%. Total protein content was observed in the range of 6–7% in green seaweeds, 3–7% in brown (except STO) and 2–4% in red seaweeds (except GRA). However, the highest content (10 ± 1%) was detected in the brown seaweed STO, while the lowest (2 ± 0.1%) was observed in the red seaweed SCI. Low lipid content < 5% was found in all seaweeds except the brown seaweeds IYN and SPA; both had about 7% total lipid content. 

The highest fiber content was detected in green seaweeds (11–27%), followed by brown seaweeds (9–13%, except LOB, which had 18%), while the lowest content of crude fiber was found in red seaweeds (2–5%). The green seaweed ACR not only contained the highest fiber content (27 ± 0.5%) but also the highest carotenoid content (11 ± 0.01 mg L^−1^). Similar to the fiber content, the highest carotenoid content was found in green seaweeds (0.5–11 mg L^−1^), followed by brown (0.5–3 mg L^−1^), while the lowest carotenoid content was found in red seaweeds (0.2–0.7 mg L^−1^). The highest total chlorophyll was found in the red seaweed GRA (41 ± 0.35 mg L^−1^), followed by the brown seaweed SAR (34 ± 0.27 mg L^−1^) and green seaweed UL (14 ± 0.11 mg L^−1^). Other seaweeds contained total chlorophyll in the range of 1.5–13 mg L^−1^. 

The highest proline content was detected in the green seaweed *Ulva* spp. (14–17 µg mg^−1^), followed by the brown seaweed STO (15 ± 0.5 µg mg^−1^), red seaweed SOL (14 ± 1 µg mg^−1^) and brown seaweed LOB (13 ± 0.3 µg mg^−1^). Other seaweeds contained proline in the range of 2–8 µg mg^−1^. The lowest iodine content (1–5 µg g^−1^) was noticed in red seaweeds, while brown seaweeds contained the highest iodine content, followed by green seaweeds. The highest iodine content was found in the brown seaweed LOB (60 ± 6 µg g^−1^), followed by SAR (41 ± 2 µg g^−1^), SPA (33 ± 2 µg g^−1^) and STO (30 ± 0.5 µg g^−1^). Among green seaweeds, UL had the highest iodine content of 30 µg g^−1^, while others were in the range of 5–15 µg g^−1^. Most seaweeds contained 40–60% nitrogen-free extract (NFE), except AMP and IYN, which contained the lowest NFE contents of 21–25%, while HAL contained the highest at 75 ± 8%, followed by UF (66 ± 4%). 

The highest phenolic content (TPC) was measured in the green seaweed ACR (107 ± 1 mg g^−1^), followed by the brown seaweeds SAR and IYN (61 ± 1 mg g^−1^), SPA (47 ± 0.5 mg g^−1^) and STO (42 ± 0.5 mg g^−1^). All other seaweeds contained TPC in the range of 10–30 mg g^−1^. The highest total flavonoid content (TFC) was detected in the brown seaweed SPA (290 ± 4 mg g^−1^), followed by the green seaweeds ACR (277 ± 3 mg g^−1^), SAR (200 ± 18 mg g^−1^) and PAD (156 ± 10 mg g^−1^). Most seaweeds contained TFC in the range of 25–75 mg g^−1^.

High micro- and trace elements were detected in all the seaweeds abundantly available along the Saurashtra coast of the Arabian Sea (Table 2). Brown seaweeds contained the highest range of Na content (1500–11,000 mg per 100 g dry weight), while the red seaweeds contained a moderate range of Na content (1400–3000 mg per 100 g; except AMP and SOL). However, green seaweeds had the lowest range of Na content (600–2200 mg per 100 g).

Red seaweeds had the highest K content, followed by brown and green seaweeds. The highest K content of 25,200 ± 220 mg per 100 g was found in SCI, followed by GRA (22,350 ± 300 mg per 100 g) and SOL (16,800 ± 200 mg per 100 g). Green seaweeds contained 3000–9000 mg per 100 g K content, while brown seaweeds contained 5300–16,000 mg per 100 g K content. Potassium content of the brown seaweed SPA (16,000 ± 400 mg per 100 g) was comparable to that of the red seaweed SOL. The red seaweed AMP contained the lowest K content (860 ± 90 mg per 100 g) among all seaweeds. Similarly, the highest Mg content was detected in red seaweeds (1400–5000 mg per 100 g), followed by green (1400–4800 mg per 100 g, except CAU) and brown (1000–2000 mg per 100 g, except PAD) seaweeds. The highest Mg content was detected in the red seaweed AMP (5000 ± 50 mg per 100 g), followed by the green seaweeds UF and UL. The magnesium content of the brown seaweed PAD and red seaweed SCI were comparable, while the lowest Mg content was detected in the green seaweed CAU (800 ± 100 mg per 100 g), followed by the brown seaweed SAR (900 ± 75 mg per 100 g). The maximum Ca content was detected in the red seaweed AMP (3500 ± 50 mg per 100 g); however, other red seaweeds contained lower ranges (45–85 mg per 100 g) of Ca content. The highest range of Ca content was detected in brown seaweeds (160–820 mg per 100 g), followed by green seaweeds (50–270 mg per 100 g).

The highest concentration of iron (36 ± 10 mg per 100 g) was found in the red seaweed AMP, followed by the brown seaweed IYN (6 ± 0.1 mg per 100 g) and green seaweed ACR (2 ± 0.01 mg per 100 g); however, all other seaweeds contained iron in the range of 0.1–1.0 mg per 100 g dry weight. Similarly, Zn and Mn contents were detected in all seaweeds in the range of 0.1–3.0 mg per 100 g (except PAD) and 0.2–4.5 mg per 100 g (except IYN, PAD and SAR), respectively. Most of the elements were detected in traces and mostly in brown and red seaweeds; however, none were found in green seaweed, except Co in ACR. 

### 3.2. Physicochemical Characterization

Seaweeds were characterized in terms of the water-swelling capacity (WSC) and water- and oil-holding capacities (Table 3). The highest WSC of 22 ± 5 mL g^−1^ dry weight (DW) was observed for the red seaweed HAL, followed by GRA (7 ± 1 mL g^−1^) and SOL (6 ± 0.5 mL g^−1^), respectively. All other seaweeds showed WSCs in the range of 1–5 mL g^−1^ DW. The water- and oil-holding capacities were observed to be almost similar in all seaweeds and in ranges of 6–8 and 7–9 g g^−1^ DW, respectively. 

### 3.3. Oxidation Stability of Oil from Seaweed Supplementation under Accelerated Conditions

The oxidative stability of peanut oil resulting from different seaweeds and BHT (standard synthetic antioxidant) was studied for various accelerated storage condition times (0–15 days) by determining the peroxide value (PV), p-anisidine value (AV), conjugated dienes (CDs), total oxidation (TOTOX) and thiobarbituric acid-reactive substances (TBARS). 

The PV values were found to be lower than with the BHT (468 mEq O_2_ kg^−1^) at the 15th day for all seaweeds (Table 4). The lowest value was observed for the green seaweed ACR (152 mEq O_2_ kg^−1^), followed by the brown seaweed SAR (224 mEq O_2_ kg^−1^). For all other seaweeds, PV values ranged from 270 to 400 mEq O_2_ kg^−1^ (except HAL: 472 mEq O_2_ kg^−1^), while a 568 mEq O_2_ kg^−1^ PV was noticed for oil kept without any supplementation (control). Similarly, lower PA values were found (Table 5) for all seaweeds (36–47) compared to BHT (47) and the control (56), with the lowest PA values found for ACR (36) followed by SAR (40).

The CDs (Table 6), TOTOX (Table 7) and TBARS (Table 8) values were also found to be lower in seaweed supplementations compared to the standard BHT and the control (crude without any antioxidant supplementation) at the end of storage (day 15). Similarly to the above, among all seaweeds, the green seaweed ACR showed the lowest values for all parameters (except TBARS) used to determine the oxidative stability of peanut oil. CDs values ranged from 4.5 to 5.2% for seaweeds, which was lower than for BHT (6.7%) and the control (7%). TOTOX values for the control and BHT were 1190 and 984, respectively, while they were 340–885 for all seaweeds except HAL, which showed a TOTOX value of 991, more than BHT. The lowest TOTOX value of 340 was found for ACR, followed by SAR (488). Surprisingly, the lowest TBARS value (0.31 mg kg^−1^) was determined for oil supplemented with the red seaweed AMP, followed by the green seaweed UF (0.32 mg kg^−1^) and ACR (0.33 mg kg^−1^), at day 15 of the accelerated storage condition. All other seaweeds showed 0.34–0.36 mg kg^−1^ TBARS, lower than the standard BHT (0.39 mg kg^−1^) and the control (0.45 mg kg^−1^).

### 3.4. Biological Activity

The cation scavenging, free radical scavenging and total reducing activities of seaweed powders (freeze-dried) were determined by using ABTS and DPPH radicals and ferric ions, respectively, on the basis of the concentration gradient (Supplementary Figures S1–S3).

The green seaweed ACR and brown seaweeds SAR and IYN showed more than 50% of the total antioxidant (ABTS radical inhibition) at their lowest concentration (<1 mg) (Table 9). Approximately 90% inhibition was determined for ACR and IYN, while SAR had almost 70% inhibition capability at the lowest concentration (1 mg). At the highest concentration of seaweed powder (5 mg), 10 seaweeds (UL, ACR, SAR, SPA, IYN, STO, LOB, SCI, AMP and GRA) showed inhibition activity above 50% (Supplementary Figure S1). The highest EC_50_ dose, or lowest antioxidant activity, was observed for CAU and Hal, followed by SOL.

For the free radical (DPPH) scavenging activity (Supplementary Figure S2), ACR showed approximately 60% inhibition at a 4 mg dose, and lower values were found for UL, STO, SAR and CAU. The highest inhibition activity (60%) was shown by ACR, STO and SAR at a 5 mg dose, whereas, at a similar 5 mg dose, UL, CAU and SPA showed almost 50–55% scavenging activity. The lowest activity, or highest EC_50_ dose, was found for HAL (17%; EC_50_ 27 mg mL^−1^), followed by AMP (19%; EC_50_ 21 mg mL^−1^) and SOL (19%; EC_50_ 12 mg mL^−1^). The lowest EC_50_ dose was found for UL (1 mg mL^−1^), followed by SAR (1 mg mL^−1^), ACR (2 mg mL^−1^), STO (3 mg mL^−1^) and CAU (4 mg mL^−1^) (Table 9).

The highest reducing capacity (Supplementary Figure S3) was found with 5 mg of ACR (90%), followed by 5 mg of SPA, SAR, STO and IYN (75%, 70%, 60% and 59%, respectively). The lowest inhibition activity (4–5%) was observed with AMP, SOL and HAL. The lowest EC_50_ dose was detected for the green seaweed ACR (1.5 mg mL^−1^), followed by the brown seaweeds SAR, SPA and STO (3–4 mg mL^−1^) (Table 9). Other seaweeds had high EC_50_ doses and, therefore, showed lower reducing activities (Table 9).

### 3.5. Antinutritional Activity

Tannin, phytic acid, saponins, alkaloids and terpenoids were considered antinutritional compounds, and their presence in seaweeds was estimated (Table 10). Tannin was detected in negligible amounts (2–6 µg g^−1^) in seven seaweeds, with the lowest concentration of 1.5 ± 0.5 µg g^−1^ in LOB and the highest of 6 ± 0.5 µg g^−1^ in STO. Phytic acid was also detected in negligible amounts in seven seaweeds, with the highest concentration of 24 ± 0.5 µg g^−1^ in SCI, followed by CAU (12 ± 0.5 µg g^−1^), whereas SAR, SPA, STO and SOL contained phytic acid in low concentrations (2–7 µg g^−1^). Other antinutritional compounds, including saponins, alkaloids and terpenoids, were not detected in any of the seaweeds (Supplementary Figure S4). The occurrence of persistent foam was used to prove the presence of saponins, but it was absent in all 15 seaweeds, indicating the absence or the presence of negligible amounts of saponins. Alkaloids were determined from the presence of orange-red precipitate in the solution. The study of the 15 abundantly available tropical seaweeds showed that there was no colored precipitate, proving the absence of alkaloids (or non-detectable content). Similarly, the presence of terpenoids was determined by a reddish-brown color, and the results confirmed the absence (or the presence of negligible amounts) of terpenoids.

## 4. Discussion

Seaweeds are well-known as a good source of healthy food for human and/or animal consumption, and their nutritional composition and mineral composition depend on seawater area. Therefore, in this study, a range of parameters, including proximate compositions (total sugar, total protein, total lipid, moisture content, ash content, crude fiber, total carotenoid, total chlorophyll content, proline content, minerals, iodine content, total phenolics and flavonoids; Table 1 and Table 2), physicochemical characteristics (water-holding capacity, oil-holding capacity and water-swelling capacity; Table 3), antioxidant activities (ABTS, DPPH and FRAP; Table 9), lipid oxidation (PV, AV, TOTOX, TBARS and CDs value; Table 4, Table 5, Table 6, Table 7 and Table 8) and antinutritional compounds (Table 10), for 15 abundantly available tropical species from three genera (four green, six brown and five red) from the Saurashtra coast of the Arabian Sea were evaluated in terms of these seaweeds’ potential for exploration as animal feed, functional food, dietary supplements and/or natural antioxidant additives.

Moisture content affects food stability and is a key factor for the preservation of various products [31]. The moisture content of the studied seaweeds was found to be much higher than the edible Spanish seaweeds *Himanthalia elongata*, *Bifurcaria bifurcata*, *Laminaria saccharina*, *Mastocarpus stellatus* and *Gigartina pistillata* (6 to 9%) [55]. In this study, moisture content ranged from 9 to 25%; however, a similar range (10–25%) was also reported by Syad et al. [31] for *Gelidiella acerosa* and *Sargassum wightii* collected from the intertidal region of the Gulf of Mannar (India). High ash content was observed in marine macroalgae in comparison to terrestrial plants, in which the ash content ranges from 5 to 10%, except some vegetables, such as spinach [56,57]. A comparison of the results for ash content with previous studies could not be provided because it is heavily affected by the size and age of the sample, habitat condition, temperature and protocol duration [58]. In the present study, low ash content (15–27%) was found in green seaweeds, and a moderate range of ash content (24–50%) was observed in brown and red seaweeds, except AMP and HAL. The highest ash content (71%) was detected in AMP. High ash content was observed in tropical seaweeds from the Arabian coast compared to previously reported data for *Enteromorpha clathrata*, *Gracilaria corticata* and *Sargassum linearifolium* from the Red Sea coast near Massawa, Eritrea [59]. A low range of ash content (5–30%) was detected in selected edible seaweeds from Semporna, Sabah, Malaysia [60]. A high range of ash content (25–40%) was found in edible Spanish seaweeds [55].

The metabolic processes of all organisms use carbohydrates as one of the most important components. Carbohydrates are also a source of energy for respiration and other vital mechanisms. Total sugar content in seaweeds varies based on multiple factors, such as environmental conditions, species, location, etc. In this study, it ranged from almost 9 to 55% of the dry weight, which was in concordance with previous reports of 4 to 75% [61,62]. Compared to the present study, higher total sugar content (25–70%) was observed in the selected edible seaweeds from Semporna, Sabah, Malaysia [60]. Comparable sugar content (25–30%) was found in selected seaweeds from the Red Sea coast near Massawa, Eritrea [59]. In the study, the highest sugar content was detected in *Ulva lactuca* (56%), which is widely considered an edible seaweed.

Protein content depends on seaweed species, geographical area and environmental conditions. Previously, protein content was measured as 5–15% of dry weight in brown seaweeds and 10–30% of dry weight in green and red seaweeds [63]. In this study, total protein content ranged from 3 to 7% of dry weight, which was lower than the previous reports [60,63]. A total of 15 edible seaweeds from Semporna, Sabah, Malaysia, showed high ranges (5–17% of dry weight) of protein [60]. Similarly, several edible seaweeds from the northwestern Spanish coast showed high ranges (10–25%) of total protein content [55].

The lipid content of seaweeds is generally found to be lower than 5% of dry weight, and in this study, similar results of between 0.5 and 7% of dry weight were estimated [64]. Similarly, a low range of lipid content (0.2–1.5%) was observed in several seaweeds from the Red Sea coast near Massawa, Eritrea [59], and in 15 edible seaweeds from Semporna, Sabah, Malaysia [60].

Utilization of foods that contain high fiber protects individuals from various chronic diseases, such as colon cancer [65]. In the present study, the crude fiber content of seaweeds ranged from 2% to 27%, and a similar range was also determined in previous reports [60]. A comparable range of crude fiber content (5–20%) was observed in several seaweeds from the Red Sea coast near Massawa, Eritrea [59], and Semporna, Sabah, Malaysia [60]. Chlorophyll and proline contents have also become important nutrient elements of food.

Several Spanish seaweeds have shown new chlorophyll derivatives that help unravel the role of these chlorophyll derivatives in food composition and bioactive compound databases [66]. Previous studies proved that proline takes part in stress-stimulated phenolic biosynthesis and also plays an important role in stimulation of antioxidant enzyme response pathways [31]. Seaweeds are the major photosynthetic organism in marine environments. The carotenoid content found in seaweeds ranged from 0.1 mg L^−1^ to 11 mg L^−1^, which was a little higher than the previously reported data [67]. The chlorophyll content of these seaweeds ranged from 1 to 40 mgL^−1^, and it is the main pigment in seaweeds providing color. The NFE ranged from 21% to 75%, which was similar to previous reports [68].

The decomposition technique is used to determine iodine content. In this technique, potentially interfering organic compounds are destroyed through incineration in the presence of KOH, which is a strong alkali that uses air as an oxidant. In seaweeds, iodine is found in both organic and inorganic forms [69]. All organic and inorganic iodine in seaweeds is converted into non-volatile iodide (I^–^) during the alkali digestion, allowing the determination of the total iodine concentration in matrices rich in fat or carbohydrates. Iodine concentration is normally high in seaweeds compared to terrestrial plants (<1 µg g^−1^) [70]. The water-soluble iodine concentration in seaweeds ranges from 9 to 99%, depending on the seaweed species; the highest content has been estimated for brown seaweeds and the lowest for green seaweeds [71]. In this study, the iodine concentration ranged from 1 to 59 µg g^−1^, and the recommended daily intake (RDI) level for an adult is 150 µg. Brown seaweeds showed higher iodine content compared to the other seaweeds and previous reports. Overall, brown seaweeds contained high iodine compared to green and red seaweeds. Based on the iodine content, it can be recommended that consumption of seaweeds should be kept within the RDI limit of 150 µg iodine per day.

Previously, a positive impact was reported for seaweed supplementation on food emulsion stability [72]. This property is directly related to the physiochemical properties of seaweeds, including the binding ability for water molecules and the absorbance capacity for oils [73]. Commonly, the WHCs, OHCs and WSCs of seaweeds are correlated with polysaccharides and polysaccharide-linked proteins present in the cell wall [74]. In a previous study, the red seaweeds *G. corticata* and *G. edulis* showed 4.03 ± 0.39 g g^−1^ and 4.09 ± 0.32 g g^−1^ WHCs, respectively, which were higher than those of AMP and SCI but lower than the other red seaweeds in the present study [17]. The green seaweed *U. intestinalis* showed a higher WHC (9.54 ± 0.02 g g^−1^) than the green seaweeds in the present study [73]. The water-holding capacity of seaweeds makes them useful to avoid syneresis in food, decrease calories, convert food into a desirable viscosity and improve the texture of the formulated food.

The hydrophobicity of proteins plays an important role in the absorbance of oil [75,76]. In this study, the OHC was estimated to be higher than the previous reports of *G. fisheri* and *G. tenuistipitata*, which showed 1.83 ± 0.08 g g^−1^ and 2.12 ± 0.03 g g^−1^, respectively [74]. The OHC of the widely consumed cuisine seaweeds nori, wakame and kombu has been reported to be approximately 3 g g^−1^ [77], which is much lower than the seaweeds studied here. Similarly, another five cuisine seaweeds (fucus, laminaria, wakame, chondrus and nori) showed OHCs in the range of 1.22–1.67 g g^−1^ [55], much lower than the seaweeds reported in this study.

Minerals are chemical elements that are crucial for normal metabolic functioning. Definite concentrations of a wide range of minerals are required by the human body to function properly, and human health is also affected by these minerals [78]. The daily intake of defined amounts of minerals plays an important role in the prevention of nutrition-related chronic and degenerative diseases, such as cardiovascular disease, obesity and cancer. Some minerals are considered essential for the human body in specific amounts, including calcium, copper, iron, magnesium, zinc, potassium, sodium, phosphors, selenium, manganese, chromium and iodine [79]. It can be observed that many of the essential minerals and elements needed for human health are the major constituents of seaweeds. In seaweeds, minerals have been found to be 10 to 20 times higher than in terrestrial plants and account for 20–50% of the dry weight. Seaweeds take up minerals from seawater, making them a rich source of trace and micro-elements [80]. The cumulative contents of micro-elements (sodium, potassium, calcium and magnesium) found in the studied seaweeds were higher than in spinach (9679 mg per 100 g) and other vegetables, including sweet corn, potato, carrots, tomato and green peas (1347, 6015, 3276, 3429 and 1452 mg per 100 g, respectively). The total trace element contents (iron, zinc, manganese and copper) in seaweeds were also observed to be higher than in some vegetables but lower than in spinach (50.7 mg per 100 g) [56].

Polyphenol and flavonoids can donate hydrogen atoms to free radicals, resulting in the inactivation of the free radicals and transforming them into chain-breaking antioxidants [81]. It is evident that the antioxidant activity of seaweeds is due to combinations of different bioactive compounds [5,24,82]. The phenolic and flavonoid contents of four *Ulva* species including *U. fasciata* and *U. lactuca*, showed positive correlations with the antioxidant activity and thus negatively correlated with their EC_50_ doses [83]. Three variants—namely, gaint, tambalanghijau and green flower—of the red seaweed *Kappaphycus alvarezii* were studied for their bioactivity. It was found that gaint (commonly known as white seaweed) had the highest phenolic and flavonoid contents, which also showed positive correlations with the antioxidant activity [84]. In the present study, ACR, IYN, SAR, SPA and STO contained high phenolic contents, whereas high flavonoid contents were found in SPA, ACR, SAR, PAD and STO. The phenolic and flavonoid contents of these seaweeds showed positive correlations with antioxidant activities (ABTS, DPPH and FRAP) and thus required low EC_50_ doses. The present study is similar to the previous reports, in which phenolic and flavonoid contents showed strong positive correlations with antioxidant activities [85]. In a previous report, the flavonoid contents of *U. lactuca* and *Sargassum wightii* (1.35 ± 0.04 and 2.02 ± 0.07 mg GAE g^−1^, respectively) showed a direct relation with their antioxidant activities; these results support the strong correlation between bioactive compounds and antioxidant activity [86]. Our previous study on 18 abundant tropical seaweeds (7 green, 4 brown and 7 red) from the Saurashtra coast of the Arabian Sea confirmed that these seaweeds are rich in phenolic and flavonoid contents [25], and these contents are directly proportional to the different bioactivities and, thus, inversely proportional to EC_50_ doses [24,26,49].

In an oxidation reaction, oxygen reacts with a substance, which results in the synthesis of free radicals and other dangerous chemical compounds. Generally, oxidation is more sensitive in unsaturated fats, whereas saturated fats are comparatively resistant to oxidation. In polyunsaturated fats, the number of unstable double bonds is high and, therefore, fats are susceptible to oxidation. These fats can be oxidized by exposure to sunlight, air or moisture and even by simple heating. Peanut oil contains high amounts of polyunsaturated fatty acids, and the oil is commonly used at high temperatures to fry food items and is susceptible to oxidation. Peanut oil oxidation generates free radicals that may contribute to many diseases, such as cancer, heart diseases and premature aging [87,88,89].

Many natural antioxidants are useful for the reduction of lipid oxidation in lipid-enriched foods and increase their lifespan. Seaweed extracts have shown reductions in the oxidation of oil [90,91]. In a previous study, it was reported that four seaweeds—two brown (*Macrocystis pyrifera* and *Ecklonia radiate*) and two red (*Porphyra* sp. and *Champia* sp.)—had the ability to reduce the oxidation of fish oil [92]. Extracts of three seaweeds, *Bifurcaria bifurcate*, *Ascophyllum nodosum* and *Fucus vesiculosus*, significantly reduced the oxidation of canola oil, more so than the synthetic antioxidant BHT [21]. In the present study, supplementation with seaweed extracts was compared with the FDA-approved synthetic antioxidant BHT, and the results showed that the seaweeds had a better reduction capacity than the synthetic antioxidant, even at higher temperatures.

Addition of the synthetic antioxidant BHT reduced oxidation by about 18%, while supplementation with ACR, SAR and IYN reduced oil oxidation by about 70%, 60% and 50%, respectively. However, with supplementation with other seaweeds, 30–50% reductions were noticed, except HAL, which reduced oil oxidation by about 17–18% (similarly to BHT). The seaweeds ACR, SAR and IYN contained high phenolic and flavonoid contents and, therefore, showed high oxidation-inhibition activities compared to the other seaweeds. The results confirmed that seaweeds can be used to improve the biological value and oxidative stability of food oils, especially peanut oil.

The absence of alkaloids, terpenoids and saponins, and the presence of negligible or trace amounts of phytic acid and tannic acid, confirmed that the studied seaweeds have potential to be explored as nutritional food supplements. Saponins affect health and metabolism because of the inhibitory activity of a number of digestive enzymes, including chymotrypsin, lipase, glucosidase, trypsin and amylase, and inhibition of these enzymes results in health disorders related to indigestion [93,94,95,96]. In the present study, saponins were not detected in the studied seaweeds; however, 13–17% saponin contents have been reported by other researchers in different seaweeds [97]. Alkaloids are phytochemicals that are harmful to organisms in high concentrations because they interrupt the electrochemical transmissions of the nervous system. They are considered harmless at low concentrations. Similarly, tannic acid and phytic acid were not detected (or detected in trace amounts) in the studied seaweeds, and such values were considerably lower than the previously reported contents in different seaweeds [98].

Nutritionally, 155–230 calories per 100 g of dry seaweed were estimated for green seaweeds, 105–300 calories per 100 g for brown seaweed and 80–165 calories per 100 g for red seaweeds. The highest calorie value of about 300 calories per 100 g was observed for SPA, followed by SAR (275 calories per 100 g), STO (240 calories per 100 g), UF (230 calories per 100 g) and CAU (200 calories per 100 g). Red seaweed showed a low calorie intake value compared to the other seaweeds. Low-calorie seaweeds may also be explored in food science and technology to develop low-calorie food or food items.

## 5. Conclusions

The antioxidant activities of the studied seaweeds confirmed their potential for application as natural antioxidants to improve the biological value of food and the oxidative stability of food oils, especially peanut oil. Proximate composition analyses of the abundant tropical seaweeds confirmed that the green and brown seaweeds contained more calories per 100 g of dry seaweed. The green seaweeds *Ulva* and *Caulerpa*; the brown seaweeds *Spatoglossum asperum*, *Sargassum linearifolium* and *Padina boergesenii*; and the red seaweed *Scinaia carnosa* are good sources of energy. The study also confirmed the absence of antinutritional compounds and toxic mineral elements in the studied tropical seaweeds. The present study recommends green seaweeds for overall nutrition, followed by brown and red seaweeds. Overall, tropical seaweeds have high nutritional potential and may deserve further exploration in relation to dietary supplementation and food/feed consumption purposes; however, a detailed, prolonged toxicity analysis with a human and/or animal model system is needed before any conclusive recommendations can be made regarding regular daily consumption in food/feed. Furthermore, they may also be explored as key ingredients in food toppings and for garnishing or seasoning food or food products.

## Figures and Tables

**Table 1:** Proximate composition of abundantly available seaweeds from the Saurashtra coast of the Arabian Sea (Gujarat, India).

	Green Seaweeds				Brown Seaweeds						Red Seaweeds				
	ACR	CAU	UF	UL	IYN	LOB	PAD	SAR	SPA	STO	AMP	GRA	HAL	SCI	SOL
MC	19 ± 2 ^a^	20 ± 1 ^a^	27 ± 1 ^b^	25 ± 1 ^b^	18 ± 2 ^a^	22 ± 1 ^a^	14 ± 1 ^c^	14 ± 1 ^c^	21 ± 2 ^a^	20 ± 1 ^a^	9 ± 1 ^d^	21 ± 1 ^a^	19 ± 1 ^a^	21 ± 2 ^a^	22 ± 2 ^a^
AC	22 ± 2 ^a^	15 ± 2 ^b^	27 ± 1 ^b^	16 ± 2 ^b^	51 ± 2 ^d^	24 ± 2 ^a^	32 ± 2 ^e^	24 ± 2 ^a^	32 ± 1 ^e^	29 ± 2 ^e^	71 ± 2 ^f^	39 ± 2 ^g^	17 ± 1 ^b^	46 ± 2 ^h^	42 ± 2 ^h^
TS	16 ± 2 ^a,c^	23 ± 2 ^b^	36 ± 1 ^d^	56 ± 2 ^e^	9 ± 1 ^f^	12 ± 2 ^c,f^	26 ± 1 ^b^	53 ± 3 ^e^	49 ± 1 ^e^	39 ± 2 ^d^	9 ± 1 ^f^	12 ± 1 ^c^	22 ± 1 ^b^	30 ± 1 ^g^	18 ± 1 ^a^
TP	7 ±1 ^a^	6 ± 1 ^a,c^	7 ± 1 ^a^	6 ± 1 ^a,c^	5 ± 1 ^c,d^	3 ± 0.4 ^b^	6 ± 1 ^a,c^	7 ± 1 ^a^	5 ± 1 ^c,d^	10 ± 1 ^e^	3 ± 0.5 ^b^	7 ± 1 ^a^	3 ± 1 ^b^	2 ± 0.1 ^f^	4 ± 1 ^b,d^
TL	3.0 ± 0.2 ^a^	4.0 ± 0.5 ^b^	4.0 ± 0.5 ^b^	1.0 ± 0.5 ^c^	7.0 ± 0.5 ^d^	1.0 ± 0.3 ^c^	5.0 ± 0.6 ^b^	1.0 ± 0.3 ^c^	7.0 ± 0.8 ^d^	2.0 ± 0.3 ^e^	3.0 ± 0.1 ^a^	2.0 ± 0.5 ^e^	1.0 ± 0.4 ^c^	3.0 ± 1.0 ^a^	2.0 ± 0.6 ^c,e^
CF	27.0 ± 0.5 ^a^	24.0 ± 2.2 ^b^	11.0 ± 0.1 ^c^	11.0 ± 0.9 ^c^	9.0 ± 0.2 ^d^	18.0 ± 0.5 ^e^	11.0 ± 0.5 ^c^	12.0 ± 0.5 ^f^	13.0 ± 0.8 ^f^	13.0± 0.7 ^f^	2.0 ± 0.2 ^g^	2.0 ± 0.1 ^g^	3.0 ± 0.2 ^h^	5.0 ± 0.5 ^i^	4.0 ± 0.1 ^j^
CC	11.00 ± 0.01 ^a^	8.00 ± 0.01 ^b^	0.50 ± 0.01 ^c^	0.80 ± 0.02 ^g^	0.70 ± 0.01 ^i^	1.40 ± 0.01 ^d^	0.50 ± 0.01 ^c^	3.0 ± 0.1 ^e^	1.00 ± 0.01 ^d^	2.00 ± 0.01 ^f^	0.30 ± 0.01 ^e^	0.30 ± 0.08 ^e^	0.20 ± 0.01 ^h^	0.70 ± 0.01 ^i^	0.20 ± 0.01 ^h^
TC	7.00 ± 0.05 ^a^	3.00 ± 0.05 ^d,e^	10.00 ± 0.04 ^g^	14.00 ± 0.11 ^h^	1.50 ± 0.02 ^f^	1.50 ± 0.01 ^f^	4.00 ± 0.05 ^c,d^	34.00 ± 0.27 ^j^	2.50 ± 0.05 ^e^	5.00 ± 0.02 ^b,c^	13.00 ± 0.15 ^i^	41.00 ± 0.35 ^k^	7.00 ± 0.05 ^a^	1.50 ± 0.01 ^f^	6.00 ± 0.05 ^a,b^
Pro	3.0 ± 0.1 ^a^	5.0 ± 0.1 ^b^	17.0 ± 0.5 ^c^	14.0 ± 4.5 ^c,d,e,f^	6.0 ± 0.2 ^g^	13.0 ± 0.3 ^d^	11.0 ± 0.5 ^e^	8.0 ± 0.2 ^h^	2.0 ± 0.3 ^i^	15.0 ± 0.5 ^f^	2.0 ± 0.2 ^i^	6.0 ± 0.1 ^g^	5.0 ± 0.1 ^b^	11.0 ± 0.3 ^e^	14.0 ± 0.7 ^d,f^
I	15 ± 1 ^a^	4 ± 1 ^b^	9 ± 1 ^c^	30 ± 11 ^d,f^	8 ± 1 ^e^	60 ± 6 ^g^	8 ± 1 ^e^	41 ± 2 ^d^	33 ± 2 ^f^	30 ± 1 ^f^	5 ± 1 ^b^	1.0 ± 0.1 ^h^	2.0 ± 0.1 ^i^	2.0 ± 0.3 ^i^	4 ± 1 ^b^
NFE	41 ± 3 ^a^	51 ± 2 ^b^	51 ± 3 ^b^	66 ± 4 ^c^	26 ± 2 ^d^	54 ± 4 ^b^	46 ± 6 ^a,b^	56 ± 3 ^b^	43 ± 5 ^a^	46 ± 3 ^a,b^	21 ± 3 ^d^	50 ± 5 ^a,b^	75 ± 8 ^c^	45 ± 8 ^a^	48 ± 4 ^a,b^
TPC	107 ± 1 ^a^	26 ± 1 ^b^	23 ± 4 ^b,c^	22 ± 1 ^c^	61 ± 1 ^d^	9 ± 1 ^e^	24 ± 1 ^b,c^	61 ± 1 ^d^	47 ± 1 ^f^	42 ± 1 ^g^	5 ± 1 ^h^	23 ± 1 ^b,c^	10 ± 1 ^e^	31 ± 1 ^i^	23 ± 2 ^b,c^
TFC	277 ± 3 ^a^	25 ± 5 ^f^	54 ± 10 ^h,i,k^	56 ± 9 ^h,k^	39 ± 4 ^i^	74 ± 16 ^h,j^	156 ± 10 ^b^	200 ± 18 ^c^	290 ± 4 ^d^	128 ± 2 ^e^	41 ± 6 ^k^	74 ± 14 ^h,j^	18 ± 1 ^g^	95 ± 15 ^j^	19 ± 6 ^f,g^
Cal.	173 ± 12 ^a,d^	200 ± 18 ^a,b^	230 ± 10 ^b^	155 ± 15 ^d^	137 ± 7 ^e^	105 ± 13 ^c^	195 ± 12 ^a^	273 ± 16 ^g^	305 ± 15 ^g^	240 ± 13 ^e^	79 ± 7 ^f^	98 ± 8 ^c^	115 ± 8 ^c^	165 ± 12 ^d^	114 ± 11 ^c^

MC: moisture content (%); AC: ash content (%); TS: total sugar (%); TP: total protein (%); TL: total lipid (%); CF: crude fiber (%); CC: carotenoid content (mg L^−1^); TC: total chlorophyll (mg L^−1^); Pro: proline (µg mg^−1^); I: iodine content (µg g^−1^); NFE: nitrogen-free extract (%); TPC: total phenolic content (mg g^−1^); TFC: total flavonoid content (mg g^−1^); Cal.: claoric content (calories per 100 g dry seaweeds). Data are mean values of triplicate samples ± SE, and different letters indicate a statistically significant difference (Tukey test *p* < 0.05). ACR: *Acrosiphonia orientalis*; CAU: *Caulerpa scalpelliformis*; UF: *Ulva fasciata*; UL: *Ulva lactuca*; IYN: *Iyengaria stellata*; LOB: *Lobophora variegata*; PAD: *Padina boergesenii*; SAR: *Sargassum linearifolium*; SPA: *Spatoglossum asperum*; STO: *Stoechospermum marginatum*; AMP: *Amphiroa anceps*; GRA: *Grateloupia indica*; HAL: *Halymenia porphyriformis*; SCI: *Scinaia carnosa*; SOL: *Solieria chordalis.*

**Table 2:** Mineral/element composition of abundantly available seaweeds from the Saurashtra coast of the Arabian Sea (Gujarat, India).

	Green Seaweeds				Brown Seaweeds						Red Seaweeds				
	ACR	CAU	UF	UL	IYN	LOB	PAD	SAR	SPA	STO	AMP	GRA	HAL	SCI	SOL
Microelements															
Na	1400 ± 125 ^a^	600 ± 110 ^b^	2200 ± 100 ^c^	2000 ± 100 ^c^	11,000 ± 250 ^d^	6800 ± 120 ^e^	5300 ± 120 ^f^	7000 ± 130 ^e^	1500 ± 170 ^g^	5500 ± 270 ^f^	275 ± 20 ^h^	2800 ± 50 ^i^	2700 ± 30 ^i^	1400 ± 70 ^a^	7400 ± 50 ^j^
K	4400 ± 120 ^a^	9300 ± 250 ^b^	8800 ± 300 ^b^	3000 ± 220 ^c^	11,700 ± 400 ^d^	7250 ± 140 ^e^	5300 ± 260 ^f^	6800 ± 190 ^g^	16,000 ± 400 ^h^	11,300 ± 260 ^d^	860 ± 90 ^i^	22,350 ± 300 ^j^	5800 ± 50 ^j^	25,200 ± 220 ^k^	16,800 ± 200 ^h^
Mg	1400 ± 100 ^a^	800 ± 100 ^c^	4800 ± 130 ^d^	4600 ± 130 ^d^	1700 ± 110 ^e^	1300 ± 150 ^a,b^	4000 ± 100 ^f^	900 ± 75 ^g^	1700 ± 100 ^e^	1200 ± 100 ^b^	5000 ± 50 ^h^	1400 ± 20 ^a^	2200 ± 80 ^i^	4000 ± 80 ^j^	2100 ± 80 ^i^
Ca	270 ± 30 ^a^	44 ± 7 ^b^	160 ± 30 ^d^	62 ± 20 ^b,c^	820 ± 35 ^e^	300 ± 30 ^a^	200 ± 30 ^d^	300 ± 20 ^a^	80 ± 15 ^c^	160 ± 30 ^d^	3500 ± 50 ^f^	60 ± 10 ^b,c^	85 ± 15 ^c^	70 ± 10 ^c^	45 ± 10 ^b^
Trace elements															
Fe	2.00 ± 0.01 ^a^	0.50 ± 0.01 ^b^	-	0.40 ± 0.01 ^b,c^	6.00 ± 0.12 ^d^	0.80 ± 0.04 ^e^	0.50 ± 0.01 ^b^	0.30 ± 0.01 ^c^	0.10 ± 0.01 ^f^	0.40 ± 0.02 ^b,c^	36 ± 10 ^g^	0.40 ± 0.01 ^b,c^	1.00 ± 0.01 ^h^	-	0.20 ± 0.01 ^f^
Zn	2.20 ± 0.01 ^a^	2.00 ± 0.01 ^a^	3.00 ± 0.01 ^b^	4.00 ± 0.01 ^b^	2.60 ± 1.20 ^a,b^	1.40 ± 0.13 ^c^	12.00 ± 2.45 ^e^	2.50 ± 1.10 ^a,b^	1.50 ± 0.44 ^a,c^	1.50 ± 0.69 ^a,c^	0.80 ± 0.01 ^d^	1.10 ± 0.01 ^c^	3.00 ± 0.01 ^b^	2.20 ± 0.01 ^a^	1.40 ± 0.01 ^a,c^
Cu	-	-	-	-	0.10 ± 0.05 ^a^	-	-	-	-	-	-	-	0.30 ± 0.01 ^b^	-	-
Mn	2.20 ± 0.02 ^a^	0.20 ± 0.01 ^b^	4.40 ± 0.02 ^c^	1.80 ± 0.01 ^a^	9.5 ± 2.0 ^d^	0.80 ± 0.03 ^e^	32.0 ± 3.6 ^f^	16.0 ± 3.3 ^g^	0.90 ± 0.02 ^e^	1.5 ± 0.6 ^e^	-	0.60 ± 0.01 ^e^	1.10 ± 0.02 ^e^	0.90 ± 0.01 ^e^	1.30 ± 0.01 ^e^
Co	0.30 ± 0.01 ^a^	-	-		0.20 ± 0.01 ^a^	-	0.10 ± 0.01 ^a^	0.20 ± 0.01 ^a^	0.90 ± 0.01 ^b^	-	-	-	0.10 ± 0.01 ^a^	11.80 ± 1.40 ^c^	2.70 ± 1.10 ^d^
As	-	-	-	-	-	0.90 ± 0.01 ^a^	-	1.30 ± 0.96 ^a^	-	0.40 ± 0.05 ^b^	-	0.10 ± 0.01 ^c^	0.20 ± 0.01 ^c^	-	0.10 ± 0.01 ^c^
Mo	-	-	-	-	-	-	0.40 ± 0.01 ^a^	-	-	-	-	0.20 ± 0.01 ^b^	-	0.10 ± 0.02 ^b^	-
Cd	-	-	-	-	-	-	-	0.10 ± 0.01	-	-	-	-	-	-	-
Hg/Pb	-	-	-	-	-	-	-	-	-	-	-	-	-	-	-
B	-	-	-	-	10 ± 1 ^a,b^	7 ± 1 ^b^	13 ± 6 ^a,b,c^	14 ± 2 ^a^	5 ± 1 ^d^	12 ± 2 ^a^	4 ± 1 ^d^	4 ± 1 ^d^	5 ± 1 ^d^	14 ± 3 ^a,c^	16 ± 1 ^c^
Cr	-	-	-	-	0.50 ± 0.01 ^a^	-	1.00 ± 0.91 ^a^	-	0.10 ± 0.01 ^b^	0.10 ± 0.01 ^b^	-	0.10 ± 0.01 ^b^	0.10 ± 0.01 ^b^	-	-
Ni	-	-	-	-	1.50 ± 0.44 ^a^	0.10 ± 0.01 ^b^	0.40 ± 0.01 ^c,d^	0.60 ± 0.01 ^d^	-	0.30 ± 0.01 ^c^	0.10 ± 0.01 ^b^	-	-	-	0.10 ± 0.01 ^b^
Na/K	0.3	0.07	0.3	0.7	1	1	1	1	0.1	0.5	0.3	0.1	0.5	0.05	0.4

-: not detected/negligible amount. Amounts are represented as mg per 100 g of dry weight and data are means ± standard error (SE), n = 6. Data with different letters indicate a statistically significant difference (Tukey test *p* < 0.05). ACR: *Acrosiphonia orientalis*; CAU: *Caulerpa scalpelliformis*; UF: *Ulva fasciata*; UL: *Ulva lactuca;* IYN: *Iyengaria stellata*; LOB: *Lobophora variegata*; PAD: *Padina boergesenii*; SAR: *Sargassum linearifolium*; SPA: *Spatoglossum asperum*; STO: *Stoechospermum marginatum*; AMP: *Amphiroa anceps*; GRA: *Grateloupia indica*; HAL: *Halymenia porphyriformis*; SCI: *Scinaia carnosa*; SOL: *Solieria chordalis.*

**Table 3:** Physicochemical characteristics of abundantly available seaweeds from the Saurashtra coast of the Arabian Sea (Gujarat, India).

	Green Seaweeds				Brown Seaweeds						Red Seaweeds				
	ACR	CAU	UF	UL	IYN	LOB	PAD	SAR	SPA	STO	AMP	GRA	HAL	SCI	SOL
WSC	2.0 ± 0.2 ^a,b^	1.0 ± 0.2 ^a^	5.0 ± 0.5 ^c^	3.0 ±0.3 ^b^	2.0 ± 0.2 ^a,b^	5.0 ± 0.3 ^c^	3.0 ± 0.2 ^b^	3.0 ± 0.2 ^b^	1.0 ± 0.3 ^a^	4.0 ± 0.4 ^c^	1.0 ± 0.2 ^a^	7.0 ±0.4 ^d^	22.0 ± 0.5 ^d^	1.5 ± 0.4 ^a^	6.0 ± 0.3 ^d^
WHC	8.00 ± 0.02 ^a^	7.00 ± 0.04 ^a,b^	7.00 ± 0.02 ^a,b^	6.00 ± 0.04 ^b^	6.0 ± 0.01 ^b^	6.00 ± 0.03 ^b^	7.00 ± 0.01 ^a,b^	7.00 ± 0.01 ^a,b^	6.00 ± 0.02 ^b^	6.00 ± 0.05 ^b^	5.00 ± 0.02 ^c^	6.00 ± 0.02 ^b^	6.00 ± 0.04 ^b^	6.00 ± 0.02 ^b^	6.00 ± 0.01 ^b^
OHC	8.00 ± 0.03 ^a,c^	9.00 ± 0.03 ^a^	7.00 ± 0.02 ^c^	7.00 ± 0.03 ^c^	7.00 ± 0.05 ^c^	7.00 ± 0.01 ^c^	7.00 ± 0.01 ^c^	7.00 ± 0.03 ^c^	7.00 ± 0.02 ^c^	7.00 ± 0.01 ^c^	7.00 ± 0.01 ^c^	7.00 ± 0.01 ^c^	7.00 ± 0.03 ^c^	7.00 ± 0.03 ^c^	7.00 ± 0.02 ^c^

WSC: water-swelling capacity (ml g^−1^ DW); WHC: water-holding capacity (g g^−1^ DW); OHC: oil-holding capacity (g g^−1^ DW). Data are mean values of triplicate samples ± SE, and different letters indicate a statistically significant difference (Tukey test *p* < 0.05). ACR: *Acrosiphonia orientalis*; CAU: *Caulerpa scalpelliformis*; UF: *Ulva fasciata*; UL: *Ulva lactuca*; IYN: *Iyengaria stellata*; LOB: *Lobophora variegata*; PAD: *Padina boergesenii*; SAR: *Sargassum linearifolium*; SPA: *Spatoglossum asperum*; STO: *Stoechospermum marginatum*; AMP: *Amphiroa anceps*; GRA: *Grateloupia indica*; HAL: *Halymenia porphyriformis*; SCI: *Scinaia carnosa*; SOL: *Solieria chordalis.*

**Table 4:** Effect on the peroxide value (mEq O_2_ kg^−1^) of peanut oil from the addition of tropical seaweed extracts.

	0 Days	3rd Day	6th Day	9th Day	12th Day	15th Day
Blank	10	48 ± 4 ^a,b^	220 ± 15 ^a^	244 ± 17 ^a^	392 ± 27 ^a^	568 ± 39 ^a^
BHT	10	40 ± 3 ^a^	228 ± 16 ^a^	164 ± 11 ^b^	280 ± 19 ^b^	468 ± 32 ^b^
Green seaweeds						
ACR	10	52 ± 4 ^b^	192 ± 13 ^a,b^	84 ± 16 ^c^	120 ± 8 ^c^	152 ± 10 ^c^
CAU	10	56 ± 5 ^b^	172 ± 12 ^b^	176 ± 12 ^b^	296 ± 20 ^b^	340 ± 23 ^e^
UF	10	48 ± 3 ^b^	176 ± 12 ^b^	208 ± 14 ^b^	376 ± 26 ^a^	400 ± 27 ^b^
UL	10	40 ± 2 ^a^	208 ± 14 ^a,b^	188 ± 13 ^b^	288 ± 20 ^b^	308 ± 20 ^e^
Brown seaweeds						
IYN	10	48 ± 5 ^a,b^	128 ± 9 ^c^	132 ± 9 ^d^	208 ± 14 ^d^	268 ± 17 ^f^
LOB	10	48 ± 4 ^a,b^	168 ± 11 ^b^	208 ± 14 ^b^	320 ± 22 ^b^	392 ± 23 ^b^
PAD	10	40 ± 3 ^a^	172 ± 12 ^b^	184 ± 13 ^b^	288 ± 20 ^b^	312 ± 19 ^e^
SAR	10	40 ± 2 ^a^	120 ± 8 ^c^	152 ± 10 ^b^	184 ± 13 ^d^	224 ± 14 ^g^
SPA	10	44 ± 4 ^a,b^	172 ± 11 ^b^	160 ± 12 ^b^	236 ± 16 ^e^	296 ± 19 ^e,f^
STO	10	40 ± 4 ^a^	176 ± 12 ^b^	176 ± 11 ^b^	308 ± 21 ^b^	352 ± 22 ^e^
Red seaweeds						
AMP	10	52 ± 4 ^b^	172 ± 13 ^b^	208 ± 14 ^b^	312 ± 21 ^b^	420 ± 24 ^b,d^
GRA	10	48 ± 3 ^a,b^	184 ± 13 ^b^	168 ± 11 ^b^	348 ± 24 ^b^	360 ± 21 ^e^
HAL	10	48 ± 2 ^a,b^	204 ± 14 ^a,b^	216 ± 15 ^a,b^	304 ± 21 ^b^	472 ± 23 ^d^
SCI	10	48 ± 3 ^a,b^	156 ± 11 ^b^	156 ± 11 ^b^	196 ± 13 ^d^	300 ± 18 ^e^
SOL	10	52 ± 4 ^b^	200 ± 14 ^a,b^	200 ± 12 ^b^	320 ± 20 ^b^	388 ± 16 ^b^

Peanut oil was individually mixed with extracts of 15 tropical seaweeds (500 ppm) and the control BHT (500 ppm), and samples were kept at 60 °C with continuous shaking (100 rpm) for different time ranges (0–16 days). Oil oxidation was analyzed in the terms of peroxide values during the storage of oil. ACR: *Acrosiphonia orientalis*; CAU: *Caulerpa scalpelliformis*; UF: *Ulva fasciata*; UL: *Ulva lactuca;* IYN: *Iyengaria stellata*; LOB: *Lobophora variegata*; PAD: *Padina boergesenii*; SAR: *Sargassum linearifolium*; SPA: *Spatoglossum asperum*; STO: *Stoechospermum marginatum*; AMP: *Amphiroa anceps*; GRA: *Grateloupia indica*; HAL: *Halymenia porphyriformis*; SCI: *Scinaia carnosa*; SOL: *Solieria chordalis*. Data are mean values of triplicate samples ± SE, and different letters indicate a statistically significant difference (Tukey test *p* < 0.05).

**Table 5:** Effect on the p-anisidine value of peanut oil from the addition of tropical seaweed extracts.

	0 Days	3rd Day	6th Day	9th Day	12th Day	15th Day
Blank	6.6	20.0 ± 8.3 ^a^	25.7 ± 9.8 ^a^	31.4 ± 4.2 ^a^	40.9 ± 2.4 ^a^	56.0 ± 1.3 ^a^
BHT	6.6	18.4 ± 4.5 ^a^	24.2 ± 1.3 ^a^	29.9 ± 3.5 ^a^	39.0 ±0.8 ^a^	47.4 ± 5.4 ^b^
Green seaweeds						
ACR	6.6	12.4 ± 2.4 ^a^	21.6 ± 1.8 ^a^	24.8 ± 3.4 ^a^	33.5 ± 3.9 ^a,b^	36.8 ± 2.7 ^c^
CAU	6.6	15.1 ± 2.1 ^a^	22.4 ± 1.0 ^a^	28.2 ± 4.1 ^a^	32.0 ± 5.3 ^a,b^	45.2 ± 4.6 ^b^
UF	6.6	11.0 ± 5.5 ^a^	21.8 ± 1.1 ^a^	26.0 ± 2.2 ^a^	38.7 ± 7.1 ^a,b^	44.4 ± 9.3 ^b,c^
UL	6.6	13.4 ± 2.7 ^a^	21.2 ± 5.2 ^a^	29.3 ± 3.8 ^a^	35.1 ± 5.4 ^a,b^	46.0 ± 2.0 ^b^
Brown seaweeds						
IYN	6.6	17.3 ± 1.3 ^a^	23.4 ± 4.2 ^a^	29.1 ± 5.6 ^a^	36.5 ± 1.2 ^a^	40.9 ± 1.2 ^b,c^
LOB	6.6	12.8 ± 0.4 ^a^	19.9 ± 1.2 ^a^	22.3 ± 4.5 ^a^	39.1 ± 4.8 ^a^	46.4 ± 0.4 ^b^
PAD	6.6	15.8 ± 4.7 ^a^	23.9 ± 4.1 ^a^	28.9 ± 1.1 ^a^	32.7 ± 2.7 ^a,b^	46.9 ± 6.7 ^b^
SAR	6.6	12.6 ± 0.6 ^a^	20.8 ± 2.0 ^a^	27.0 ± 2.3 ^a^	30.4 ± 2.9 ^b^	40.4 ± 1.1 ^b^
SPA	6.6	16.8 ± 5.3 ^a^	23.4 ± 6.2 ^a^	28.4 ± 2.2 ^a^	36.7 ± 5.6 ^a,b^	42.5 ± 0.8 ^b^
STO	6.6	17.1 ± 0.8 ^a^	22.3 ± 2.4 ^a^	29.0 ± 1.2 ^a^	33.6 ± 8.8 ^a,b^	44.9 ± 1.1 ^b^
Red seaweeds						
AMP	6.6	16.1 ± 2.1 ^a^	21.7 ± 3.0 ^a^	26.5 ± 4.3 ^a^	34.0 ± 5.1 ^a,b^	44.2 ± 10.4 ^b^
GRA	6.6	16.7 ± 1.7 ^a^	19.2 ± 1.3 ^a^	26.7 ± 1.4 ^a^	38.3 ± 2.4 ^a^	46.5 ± 5.3 ^b^
HAL	6.6	16.8 ± 0.5 ^a^	22.3 ± 1.4 ^a^	24.8 ± 1.8 ^a^	33.3 ± 2.6 ^a,b^	47.2 ± 5.7 ^b^
SCI	6.6	18.3 ± 4.9 ^a^	21.6 ± 1.1 ^a^	28.7 ± 2.3 ^a^	37.5 ± 11.1 ^a,b^	43.2 ± 15.6 ^b^
SOL	6.6	15.0 ± 1.5 ^a^	19.8 ± 1.4 ^a^	25.0 ± 3.0 ^a^	38.5 ± 1.5 ^a^	46.1 ± 1.2 ^b^

Peanut oil was mixed individually with extracts of 15 tropical seaweeds (500 ppm) and the control BHT (500 ppm), and samples were kept at 60 °C with continuous shaking (100 rpm) for different time ranges (0–16 days). Oil oxidation was analyzed in the terms of the p-anisidine value during the storage of oil. ACR: *Acrosiphonia orientalis*; CAU: *Caulerpa scalpelliformis*; UF: *Ulva fasciata*; UL: *Ulva lactuca;* IYN: *Iyengaria stellata*; LOB: *Lobophora variegata*; PAD: *Padina boergesenii*; SAR: *Sargassum linearifolium*; SPA: *Spatoglossum asperum*; STO: *Stoechospermum marginatum*; AMP: *Amphiroa anceps*; GRA: *Grateloupia indica*; HAL: *Halymenia porphyriformis*; SCI: *Scinaia carnosa*; SOL: *Solieria chordalis*. Data are mean values of triplicate samples ± SE, and different letters indicate a statistically significant difference (Tukey test *p* < 0.05).

**Table 6:** Effect on the conjugated dienes value (%) of peanut oil from the addition of tropical seaweed extracts.

	0 Days	3rd Day	6th Day	9th Day	12th Day	15th Day
Blank	1.23	2.08 ± 0.01 ^a^	3.69 ± 0.01 ^a^	4.19 ± 0.03 ^a^	5.58 ± 0.02 ^a^	7.00 ± 0.10 ^a^
BHT	1.23	1.83 ± 0.02 ^b^	3.28 ± 0.02 ^b^	4.02 ± 0.02 ^a^	4.58 ± 0.01 ^b^	6.68 ± 0.01 ^b^
Green seaweeds						
ACR	1.23	1.19 ± 0.02 ^c^	2.28 ± 0.02 ^c^	3.26 ± 0.02 ^b,c^	3.32 ± 0.01 ^d^	4.53 ± 0.04 ^c^
CAU	1.23	1.72 ± 0.04 ^d,g^	3.16 ± 0.02 ^e^	2.95 ± 0.03 ^e^	4.48 ± 0.03 ^e^	4.88 ± 0.09 ^d^
UF	1.23	1.73 ± 0.02 ^d^	2.78 ± 0.02 ^f^	3.27 ± 0.04 ^b^	3.85 ± 0.09 ^f,g^	4.68 ± 0.04 ^e^
UL	1.23	1.64 ± 0.01 ^e^	2.95 ± 0.01 ^g^	3.58 ± 0.01 ^f^	4.58 ± 0.02 ^b^	5.20 ± 0.03 ^f^
Brown seaweeds						
IYN	1.23	1.25 ± 0.04 ^c^	2.24 ± 0.01 ^c^	3.23 ± 0.02 ^d^	3.46 ± 0.02 ^h^	4.64 ± 0.04 ^g^
LOB	1.23	1.71 ± 0.05 ^g^	2.53 ± 0.01 ^h^	3.34 ± 0.02 ^g^	4.43 ± 0.04 ^i^	4.96 ± 0.34 ^h^
PAD	1.23	1.68 ± 0.01 ^f^	3.28 ± 0.01 ^b^	3.34 ± 0.02 ^g^	4.94 ± 0.03 ^j^	4.67 ± 0.03 ^e^
SAR	1.23	1.19 ± 0.02 ^c^	2.29 ± 0.02 ^c^	3.25 ± 0.02 ^c^	3.67 ± 0.01 ^k^	4.95 ± 0.32 ^h,i^
SPA	1.23	1.30 ± 0.03 ^h^	2.31 ± 0.01 ^c^	3.25 ± 0.01 ^c^	3.45 ± 0.02 ^h^	4.48 ± 0.03 ^j^
STO	1.23	1.77 ± 0.03 ^i^	2.87 ± 0.03 ^f^	3.40 ± 0.01 ^h^	3.97 ± 0.03 ^g^	4.73 ± 0.02 ^k^
Red seaweeds						
AMP	1.23	1.84 ± 0.02 ^b^	3.17 ± 0.03 ^e^	3.85 ± 0.01 ^i^	4.58 ± 0.07 ^b^	5.24 ± 0.03 ^f^
GRA	1.23	1.71 ± 0.01 ^g^	2.65 ± 0.01 ^h^	3.73 ± 0.03 ^j^	4.56 ± 0.03 ^c^	4.96 ± 0.05 ^h^
HAL	1.23	1.78 ± 0.01 ^i^	2.94 ± 0.04 ^g^	3.67 ± 0.07 ^k^	4.39 ± 0.04 ^l^	4.89 ± 0.02 ^d^
SCI	1.23	1.38 ± 0.05 ^k^	2.40 ± 0.02 ^i^	3.24 ± 0.02 ^d^	3.79 ± 0.01 ^f^	4.91 ± 0.01 ^l^
SOL	1.23	1.78 ± 0.03 ^i^	3.00 ± 0.02 ^g^	3.72 ± 0.02 ^l^	4.44 ± 0.02 ^i^	4.94 ± 0.03 ^i^

Peanut oil was mixed individually with extracts of 15 tropical seaweeds (500 ppm) and the control BHT (500 ppm), and samples were kept at 60 °C with continuous shaking (100 rpm) for different time ranges (0–16 days). Oil oxidation was analyzed in terms of the conjugated dienes value during the storage of oil. ACR: *Acrosiphonia orientalis*; CAU: *Caulerpa scalpelliformis*; UF: *Ulva fasciata*; UL: *Ulva lactuca;* IYN: *Iyengaria stellata*; LOB: *Lobophora variegata*; PAD: *Padina boergesenii*; SAR: *Sargassum linearifolium*; SPA: *Spatoglossum asperum*; STO: *Stoechospermum marginatum*; AMP: *Amphiroa anceps*; GRA: *Grateloupia indica*; HAL: *Halymenia porphyriformis*; SCI: *Scinaia carnosa*; SOL: *Solieria chordalis*. Data are mean values of triplicate samples ± SE, and different letters indicate a statistically significant difference (Tukey test *p* < 0.05).

**Table 7:** Effect on the TOTOX value of peanut oil from the addition of tropical seaweed extracts.

	0 Days	3rd Day	6th Day	9th Day	12th Day	15th Day
Blank	26.6	116.0 ± 8.3 ^a^	460.0 ± 9.8 ^a^	519.4 ± 4.2 ^a^	824.9 ± 2.4 ^a^	1192.0 ± 8.5 ^a^
BHT	26.6	98.4 ± 4.5 ^b,c^	480.2 ± 1.3 ^b^	357.9 ± 3.5 ^b^	599.0 ± 0.8 ^b^	983.4 ± 5.4 ^b^
Green seaweeds						
ACR	26.6	116.4 ± 2.4 ^a^	405.6 ± 1.8 ^c^	192.8 ± 3.4 ^c^	273.5 ± 3.9 ^c^	340.8 ± 2.7 ^c^
CAU	26.6	127.1 ± 2.1	366.4 ± 1.0 ^d^	380.2 ± 4.1 ^d^	624.0 ± 5.3 ^d^	725.2 ± 4.6 ^d^
UF	26.6	107.0 ± 5.5 ^b^	373.8 ± 1.1 ^e^	442.0 ± 2.2 ^e^	790.7 ± 7.1	844.4 ± 9.3 ^e^
UL	26.6	93.4 ± 2.7 ^c^	437.2 ± 5.2 ^f^	405.3 ± 3.8 ^f^	611.1 ± 5.4 ^d^	662.0 ± 2.0 ^f^
Brown seaweeds						
IYN	26.6	113.3 ± 1.3 ^a^	279.4 ± 4.2 ^g^	293.1 ± 5.6 ^g^	452.5 ± 1.2 ^e^	576.9 ± 1.2 ^g^
LOB	26.6	108.8 ± 0.4 ^a^	355.9 ± 1.2 ^h^	438.3 ± 4.5 ^e^	679.1 ± 4.8 ^f^	830.4 ± 0.4 ^e^
PAD	26.6	95.8 ± 4.7 ^c^	367.9 ± 4.1 ^d^	396.9 ± 1.1 ^f^	608.7 ± 2.7 ^b^	670.9 ± 6.7 ^f^
SAR	26.6	92.6 ± 0.6 ^c^	260.8 ± 2.0 ^g^	331.0 ± 2.3 ^h^	398.4 ± 2.9 ^g^	488.4 ± 1.1 ^h^
SPA	26.6	104.8 ± 5.3 ^b^	367.4 ± 6.2 ^d^	348.4 ± 2.2 ^k^	508.7 ± 5.6 ^h^	634.5 ± 0.8 ^f^
STO	26.6	97.1 ± 0.8 ^c^	374.3 ± 2.4 ^e^	381.0 ± 1.2 ^d^	649.6 ± 8.8 ^i^	748.9 ± 1.1 ^d^
Red seaweeds						
AMP	26.6	120.1 ± 2.1 ^a^	365.7 ± 3.0 ^d^	442.5 ± 4.3 ^e^	658.0 ± 5.1 ^i^	884.2 ± 10.4 ^e^
GRA	26.6	112.7 ± 1.7 ^a^	387.2 ± 1.3 ^i^	362.7 ± 1.4 ^i^	734.3 ± 2.4 ^j^	766.5 ± 5.3 ^d^
HAL	26.6	112.8 ± 0.5 ^a^	430.3 ± 1.4 ^f^	456.8 ± 1.8 ^j^	641.3 ± 2.6 ^i^	991.2 ± 5.7 ^b^
SCI	26.6	114.3 ± 4.9 ^a^	333.6 ± 1.1 ^j^	340.7 ± 2.3 ^k^	429.5 ± 11.1 ^e^	643.2 ± 15.6 ^f^
SOL	26.6	119.0 ± 1.5 ^a^	419.8 ± 1.4 ^k^	425.0 ± 3.0 ^l^	678.5 ± 1.5 ^f^	822.1 ± 1.2 ^e^

Peanut oil was mixed individually with extracts of 15 tropical seaweeds (500 ppm) and the control BHT (500 ppm), and samples were kept at 60 °C with continuous shaking (100 rpm) for different time ranges (0–16 days). Oil oxidation was analyzed in the terms of the TOTOX value during the storage of oil. ACR: *Acrosiphonia orientalis*; CAU: *Caulerpa scalpelliformis*; UF: *Ulva fasciata*; UL: *Ulva lactuca;* IYN: *Iyengaria stellata*; LOB: *Lobophora variegata*; PAD: *Padina boergesenii*; SAR: *Sargassum linearifolium*; SPA: *Spatoglossum asperum*; STO: *Stoechospermum marginatum*; AMP: *Amphiroa anceps*; GRA: *Grateloupia indica*; HAL: *Halymenia porphyriformis*; SCI: *Scinaia carnosa*; SOL: *Solieria chordalis*. Data are mean values of triplicate samples ± SE, and different letters indicate a statistically significant difference (Tukey test *p* < 0.05).

**Table 8:** Effect on the thiobarbituric acid-reactive substance (TBARS) value (mg kg^−1^) of peanut oil from the addition of tropical seaweed extracts.

	0 Days	3rd Day	6th Day	9th Day	12th Day	15th Day
Blank	0.009	0.049 ± 0.002 ^b^	0.078 ± 0.003 ^a^	0.276 ± 0.002 ^a^	0.367 ± 0.005 ^a^	0.454 ± 0.005 ^a^
BHT	0.009	0.044 ±0.001 ^a^	0.070 ± 0.001 ^b^	0.248 ± 0.004 ^b^	0.339 ± 0.002 ^b^	0.397 ± 0.002 ^b^
Green seaweeds						
ACR	0.009	0.017 ± 0.002 ^c^	0.045 ± 0.003 ^c^	0.150 ± 0.002 ^d^	0.281 ± 0.004 ^e^	0.333 ± 0.005 ^c^
CAU	0.009	0.037 ± 0.002 ^d^	0.070 ± 0.003 ^b^	0.243 ± 0.008 ^b^	0.321 ± 0.001 ^c^	0.350 ± 0.001 ^e^
UF	0.009	0.042 ± 0.002 ^a^	0.068 ± 0.001 ^b^	0.237 ± 0.005 ^b,c^	0.322 ± 0.002 ^c^	0.319 ± 0.017 ^c^
UL	0.009	0.036 ± 0.001 ^d^	0.068 ± 0.001 ^b^	0.241 ± 0.002 ^b^	0.315 ± 0.001 ^d^	0.361 ± 0.004 ^e^
Brown seaweeds						
IYN	0.009	0.025 ± 0.001 ^e^	0.057 ± 0.002 ^d^	0.188 ± 0.005 ^e,f^	0.312 ± 0.002 ^d^	0.345 ± 0.003 ^e^
LOB	0.009	0.037 ± 0.001 ^d^	0.073 ± 0.007 ^a,b^	0.208 ± 0.017 ^e,h^	0.338 ± 0.003 ^b^	0.367 ± 0.003 ^f^
PAD	0.009	0.039 ± 0.001 ^a,d^	0.068 ± 0.001 ^b^	0.232 ± 0.002 ^c^	0.335 ± 0.001 ^b^	0.340 ± 0.003 ^c,d^
SAR	0.009	0.022 ± 0.001	0.053 ± 0.002 ^d^	0.167 ± 0.003 ^f^	0.296 ± 0.002 ^f^	0.344 ± 0.006 ^d,e^
SPA	0.009	0.034 ± 0.001	0.050 ± 0.008 ^c,d^	0.205 ± 0.001 ^g^	0.305 ± 0.006 ^d^	0.351 ± 0.004 ^e^
STO	0.009	0.038 ± 0.003 ^a,d^	0.064 ± 0.003 ^b^	0.214 ± 0.003 ^h^	0.334 ± 0.010 ^b,c^	0.353 ± 0.001 ^e^
Red seaweeds						
AMP	0.009	0.032 ± 0.008 ^a,d^	0.067 ± 0.003 ^b^	0.214 ± 0.005 ^h^	0.331 ± 0.002 ^b^	0.308 ± 0.021 ^g^
GRA	0.009	0.035 ± 0.011 ^a,b,d^	0.065 ± 0.003 ^b^	0.212 ± 0.007 ^g,h^	0.311 ± 0.012 ^d^	0.329 ± 0.021 ^h^
HAL	0.009	0.034 ± 0.001 ^d^	0.070 ± 0.002 ^b^	0.230 ± 0.006 ^i^	0.325 ± 0.003 ^c^	0.355 ± 0.005 ^e^
SCI	0.009	0.034 ± 0.001 ^d^	0.065 ± 0.001 ^b^	0.205 ± 0.001 ^g^	0.302 ± 0.003 ^f^	0.331 ± 0.003 ^h^
SOL	0.009	0.039 ± 0.003 ^a^	0.071 ± 0.001 ^b^	0.256 ± 0.004 ^b^	0.310 ± 0.002 ^d^	0.362 ± 0.002 ^f^

Peanut oil was mixed individually with extracts of 15 tropical seaweeds (500 ppm) and the control BHT (500 ppm), and samples were kept at 60 °C with continuous shaking (100 rpm) for different time ranges (0–16 days). Oil oxidation was analyzed in the terms of the TBARS value during the storage of oil. ACR: *Acrosiphonia orientalis*; CAU: *Caulerpa scalpelliformis*; UF: *Ulva fasciata*; UL: *Ulva lactuca;* IYN: *Iyengaria stellata*; LOB: *Lobophora variegata*; PAD: *Padina boergesenii*; SAR: *Sargassum linearifolium*; SPA: *Spatoglossum asperum*; STO: *Stoechospermum marginatum*; AMP: *Amphiroa anceps*; GRA: *Grateloupia indica*; HAL: *Halymenia porphyriformis*; SCI: *Scinaia carnosa*; SOL: *Solieria chordalis*. Data are mean values of triplicate samples ± SE, and different letters indicate a statistically significant difference (Tukey test *p* < 0.05).

**Table 9:** Comparison of half-maximal effective concentration (EC_50_; mg mL^−1^) activities among abundantly available seaweeds from the Saurashtra coast of the Arabian Sea (Gujarat, India).

	Cation Scavenging Activity (ABTS)	Free Radical Scavenging Activity (DPPH)	Total Reducing Capacity (FRAP)
Green seaweeds			
ACR	<1	2.0 ± 0.1 ^a^	1.6 ± 0.1 ^a^
CAU	10.0 ± 1.5 ^a^	4.0 ± 0.1 ^b^	14.0 ± 1.0 ^b^
UF	7.0 ± 0.2 ^b^	9.0 ± 1.0 ^c,d^	36.0 ± 4.0 ^c,d^
UL	1.0 ± 0.1 ^h^	1.0 ± 0.2 ^f^	34.0 ± 0.1 ^d^
Brown seaweeds			
IYN	<1	7.0 ± 1.0 ^c^	5.0 ± 0.1 ^k^
LOB	4.00 ± 0.05 ^e^	9.0 ± 0.5 ^d^	31.0 ± 0.1 ^c^
PAD	6.0 ± 0.2 ^c^	10.0 ± 1.0 ^d,e^	31.0 ± 2.0 ^c^
SAR	<1	1.0 ± 0.5 ^f^	3.00 ± 0.05 ^e^
SPA	2.0 ± 0.2 ^g^	5.0 ± 0.1 ^g^	4.00 ± 0.05 ^f^
STO	3.00 ± 0.01 ^f^	3.0 ± 0.2 ^h^	4.00 ± 0.05 ^f^
Red seaweeds			
AMP	4.0 ± 0.1 ^e^	21.0 ± 3.0 ^i^	74.0 ± 12.0 ^g^
GRA	2.0 ± 0.1 ^g^	10.0 ± 0.1 ^d,e^	24.0 ± 0.2 ^h^
HAL	10.0 ± 0.1 ^a^	27.0 ± 3.0 ^i^	49.0 ± 1.0 ^i^
SCI	3.0 ± 0.1 ^f^	8.0 ± 1.0 ^c,d^	16.0 ± 0.5 ^b^
SOL	8.0 ± 0. 1 ^d^	12.0 ± 1.0 ^e^	45.0 ± 1.0 ^j^

ACR: *Acrosiphonia orientalis*; CAU: *Caulerpa scalpelliformis*; UF: *Ulva fasciata*; UL: *Ulva lactuca*; IYN: *Iyengaria stellata*; LOB: *Lobophora variegata*; PAD: *Padina boergesenii*; SAR: *Sargassum linearifolium*; SPA: *Spatoglossum asperum*; STO: *Stoechospermum marginatum*; AMP: *Amphiroa anceps*; GRA: *Grateloupia indica*; HAL: *Halymenia porphyriformis*; SCI: *Scinaia carnosa*; SOL: *Solieria chordalis*. Data are mean values of triplicate samples ± SE, and different letters indicate a statistically significant difference (Tukey test p < 0.05).

**Table 10:** Analysis of antinutritional compounds/activities of abundantly available seaweeds from the Saurashtra coast of the Arabian Sea (Gujarat, India).

	Green Seaweeds				Brown Seaweeds						Red Seaweeds				
	ACR	CAU	UF	UL	IYN	LOB	PAD	SAR	SPA	STO	AMP	GRA	HAL	SCI	SOL
TA	3.5 ± 0.7 ^a,c^	-	-	-	-	1.5 ± 0.5 ^b^	5 ± 1.0 ^c,d^	5 ± 0.5 ^c,d^	3.0 ± 0.3 ^a^	6.0 ± 0.5 ^d^	-	-	2.0 ± 1.0 ^a,b^	-	-
PA	-	12.0 ± 0.5 ^a^	6.0 ± 1.0 ^b,c^	-	-	-	-	5.0 ± 0.5 ^c^	7.0 ± 1.0 ^b^	2.0 ± 0.5 ^d^	-	-	-	24.0 ± 0.5 ^e^	4.0 ± 1.0 ^c^

-: not detected/no activity; TA: tannic acid (µg g^−1^); PA: phytic acid (µg g^−1^); ACR: *Acrosiphonia orientalis*; CAU: *Caulerpa scalpelliformis*; UF: *Ulva fasciata*; UL: *Ulva lactuca;* IYN: *Iyengaria stellata*; LOB: *Lobophora variegata*; PAD: *Padina boergesenii*; SAR: *Sargassum linearifolium*; SPA: *Spatoglossum asperum*; STO: *Stoechospermum marginatum*; AMP: *Amphiroa anceps*; GRA: *Grateloupia indica*; HAL: *Halymenia porphyriformis*; SCI: *Scinaia carnosa*; SOL: *Solieria chordalis*. Data are mean values of triplicate samples ± SE. Different letters indicate a statistically significant difference (*p* < 0.05).

## Data Availability

Not applicable.

## Supplementary Materials

The following supporting information can be downloaded at: https://www.mdpi.com/article/10.3390/plants12122302/s1, Table S1: Details for the abundant tropical seaweeds collected from the Saurashtra coast of the Arabian Sea, India; Figure S1: Total antioxidant activities of the abundant tropical seaweeds; Figure S2: Scavenging activities of the abundant tropical seaweeds; Figure S3: Reducing activities of the abundant tropical seaweeds; Figure S4: Estimation of the presence of antinutritional compounds in the abundant tropical seaweeds.
