# Supplementary material for: Nutrient Composition, Physicobiochemical Analyses, Oxidative Stability and Antinutritional Assessment of Abundant Tropical Seaweeds from the Arabian Sea
Source: Plants (Basel). 2023 Jun 13;12(12):2302. doi: 10.3390/plants12122302 (PMC10305345; doi:10.3390/plants12122302)
Supplement: Supplementary file 1 [file plants-12-02302-s001.zip › plants-2394114-supplementary.pdf]

Table S1: Details of abundant tropical seaweeds collected from the Saurashtra coast of the Arabian sea of India

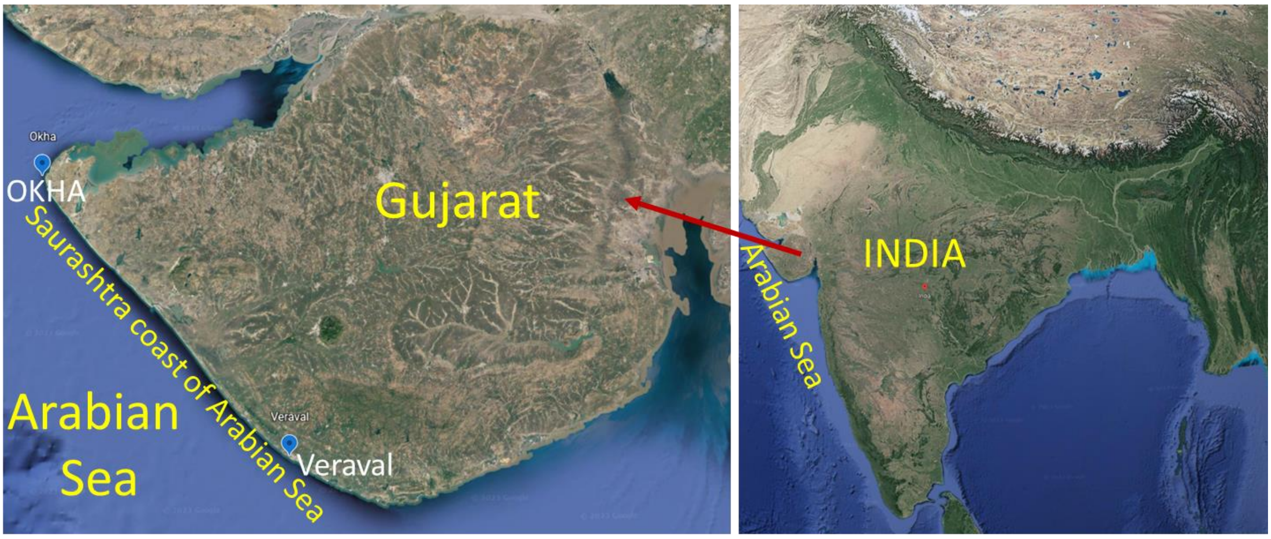

Geographical map of the sample collection site

S. No.	Seaweeds	Location	GPS coordinates	Image
**Green Seaweeds**				
1.	*Acrosiphonia orientalis*	Veraval	N. 20° 90.97'' E. 70° 35.22”	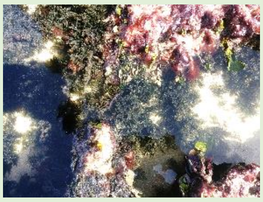
2.	*Caulerpa scalpelliformis*	Veraval	N. 20° 90.97'' E. 70° 35.22”	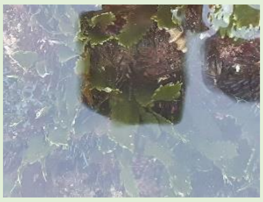
3.	*Ulva fasciata*	Veraval	N. 20° 90.97'' E. 70° 35.22”	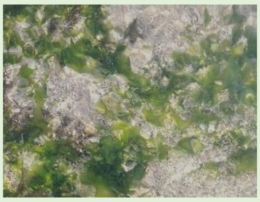
4.	*Ulva lactuca*	Veraval	N. 20° 90.97'' E. 70° 35.22”	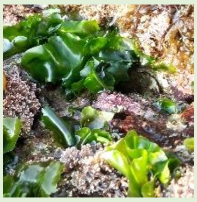
**Brown Seaweeds**				
5.	*Iyengaria stellata*	Okha	N. 22° 30.60” E. 69° 05.76”	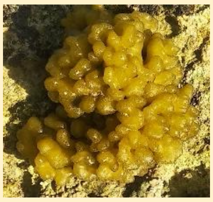
6.	*Lobophora variegata*	Veraval	N. 20° 90.97'' E. 70° 35.22”	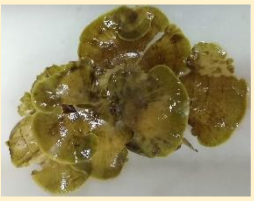

7.	*Padina boergesenii*	Okha	N. 22° 30.60”<br>E. 69° 05.76”	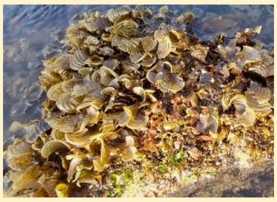
8.	*Sargassum linearifolium*	Okha	N. 22° 30.60”<br>E. 69° 05.76”	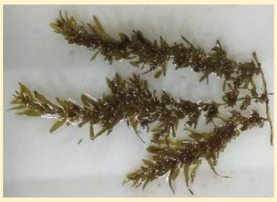
9.	*Spatoglossum asperum*	Okha	N. 22° 30.60”<br>E. 69° 05.76”	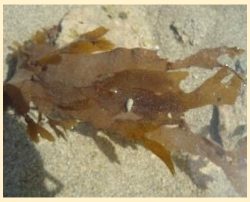
10.	*Stoechospermum marginatum*	Veraval	N. 20° 90.97’’<br>E. 70° 35.22”	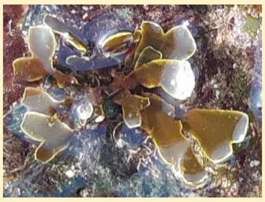
**Red Seaweeds**				
11.	*Amphiroa anceps*	Veraval	N. 20° 90.97’’<br>E. 70° 35.22”	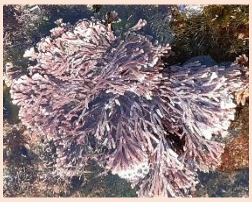
12.	*Grateloupia indica*	Veraval	N. 20° 90.97’’<br>E. 70° 35.22”	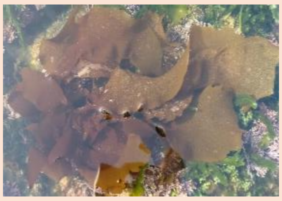
13.	*Halymenia porphyriformis*	Veraval	N. 20° 90.97’’<br>E. 70° 35.22”	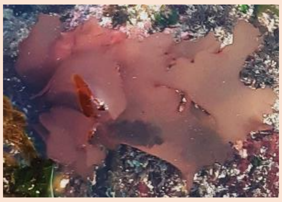
14.	*Scinaia carnosa*	Veraval	N. 20° 90.97’’<br>E. 70° 35.22”	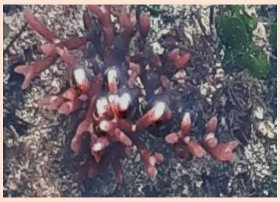
15.	*Solieria chordalis*	Veraval	N. 20° 90.97’’<br>E. 70° 35.22”	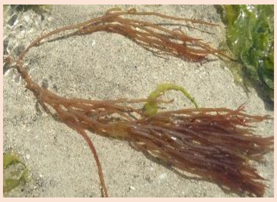

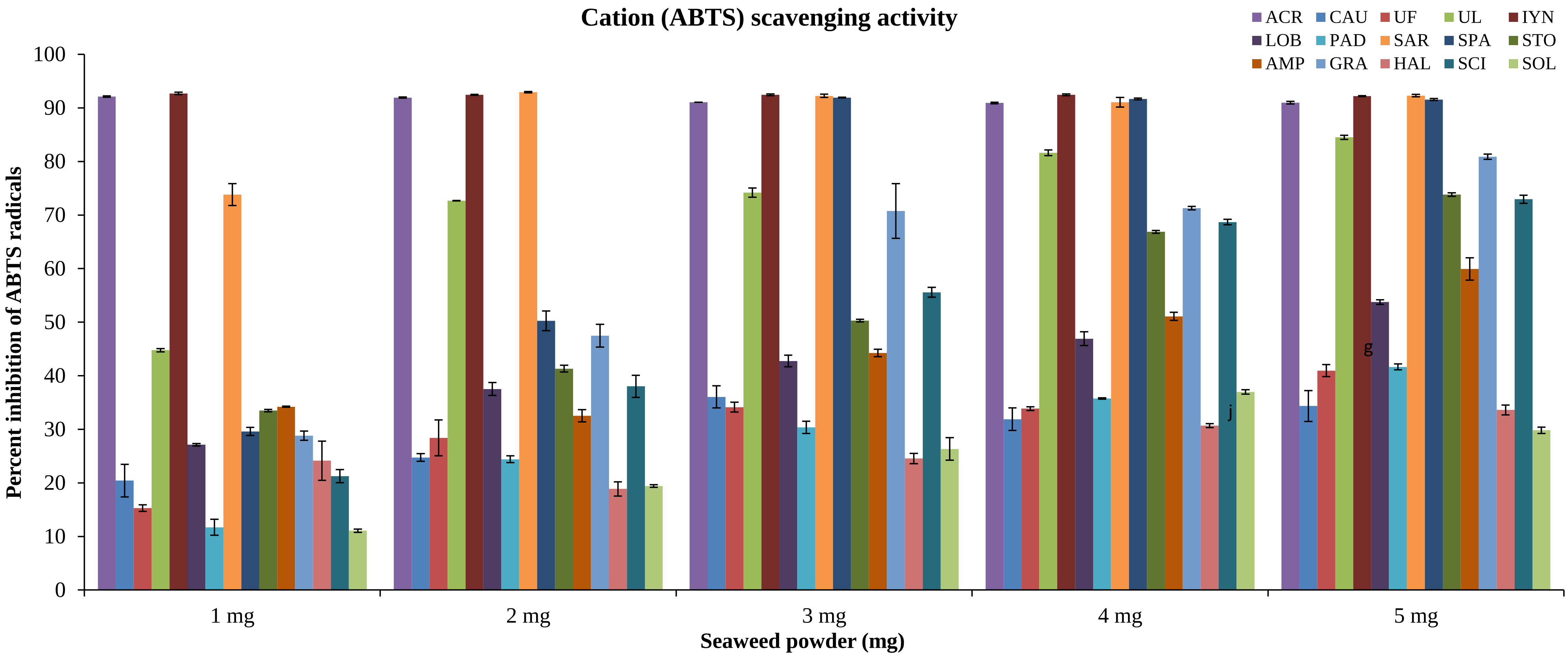

Figure S1: Total antioxidant activity of abundant tropical seaweeds. Activity is measured as percent inhibition of ABTS (2,2'-azino-bis(3-ethylbenzothiazoline-6-sulphonic acid)) free radicals, expressed as mean ± standard error of the mean (SE; n=3). ACR: *Acrosiphonia orientalis*; CAU: *Caulerpa scalpelliformis*; UF: *Ulva fasciata*; UL: *Ulva lactuca;* IYN: *Iyengaria stellata*; LOB: *Lobophora variegata*; PAD: *Padina boergesenii*; SAR: *Sargassum linearifolium*; SPA: *Spatoglossum asperum*; STO: *Stoechospermum marginatum*; AMP: *Amphiroa anceps*; GRA: *Grateloupia indica*; HAL: *Halymenia porphyriformis*; SCI: *Scinaia carnosa*; SOL: *Solieria chordalis*

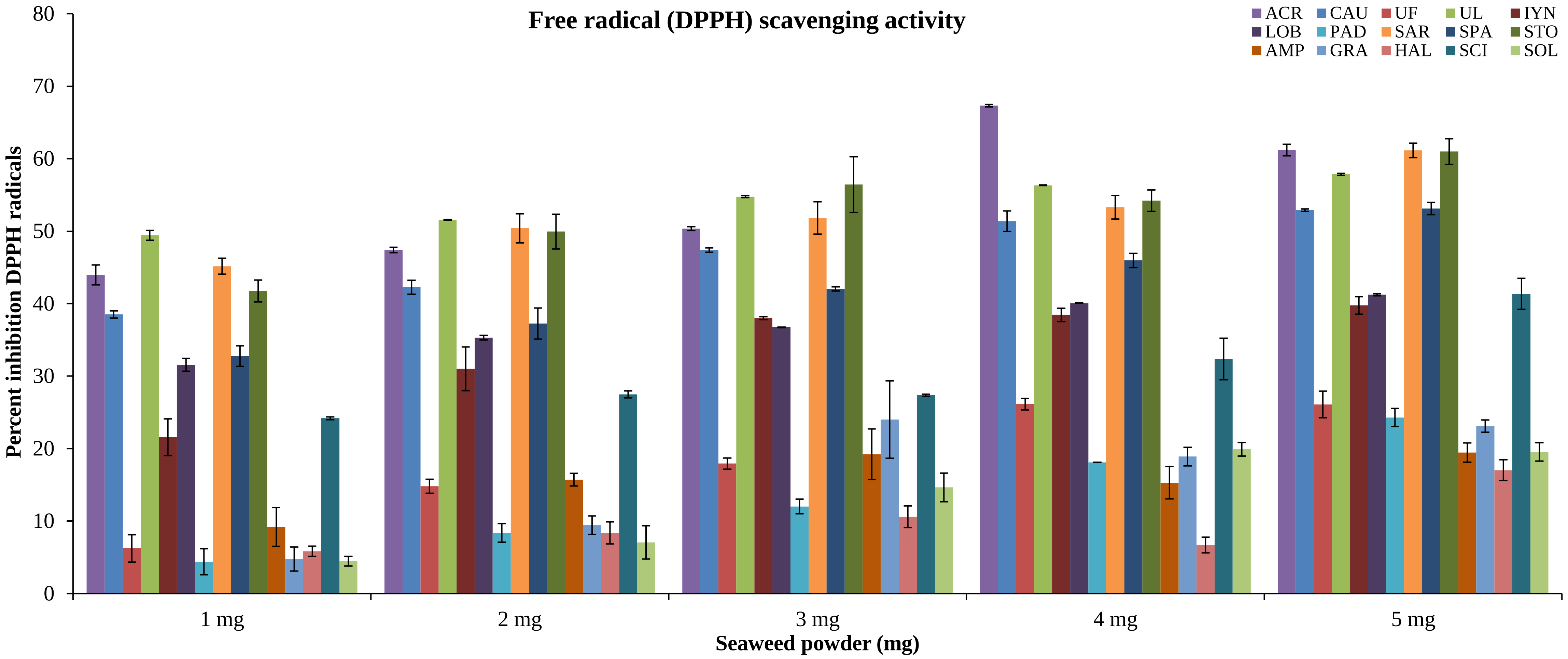

Figure S2: Scavenging activity of abundant tropical seaweeds. Activity is measured as percent inhibition of DPPH (2,2-diphenyl-1-picrylhydrazyl) free radicals, expressed as mean ± standard error of the mean (SE; n=3). ACR: *Acrosiphonia orientalis*; CAU: *Caulerpa scalpelliformis*; UF: *Ulva fasciata*; UL: *Ulva lactuca;* IYN: *Iyengaria stellata*; LOB: *Lobophora variegata*; PAD: *Padina boergesenii*; SAR: *Sargassum linearifolium*; SPA: *Spatoglossum asperum*; STO: *Stoechospermum marginatum*; AMP: *Amphiroa anceps*; GRA: *Grateloupia indica*; HAL: *Halymenia porphyriformis*; SCI: *Scinaia carnosa*; SOL: *Solieria chordalis*

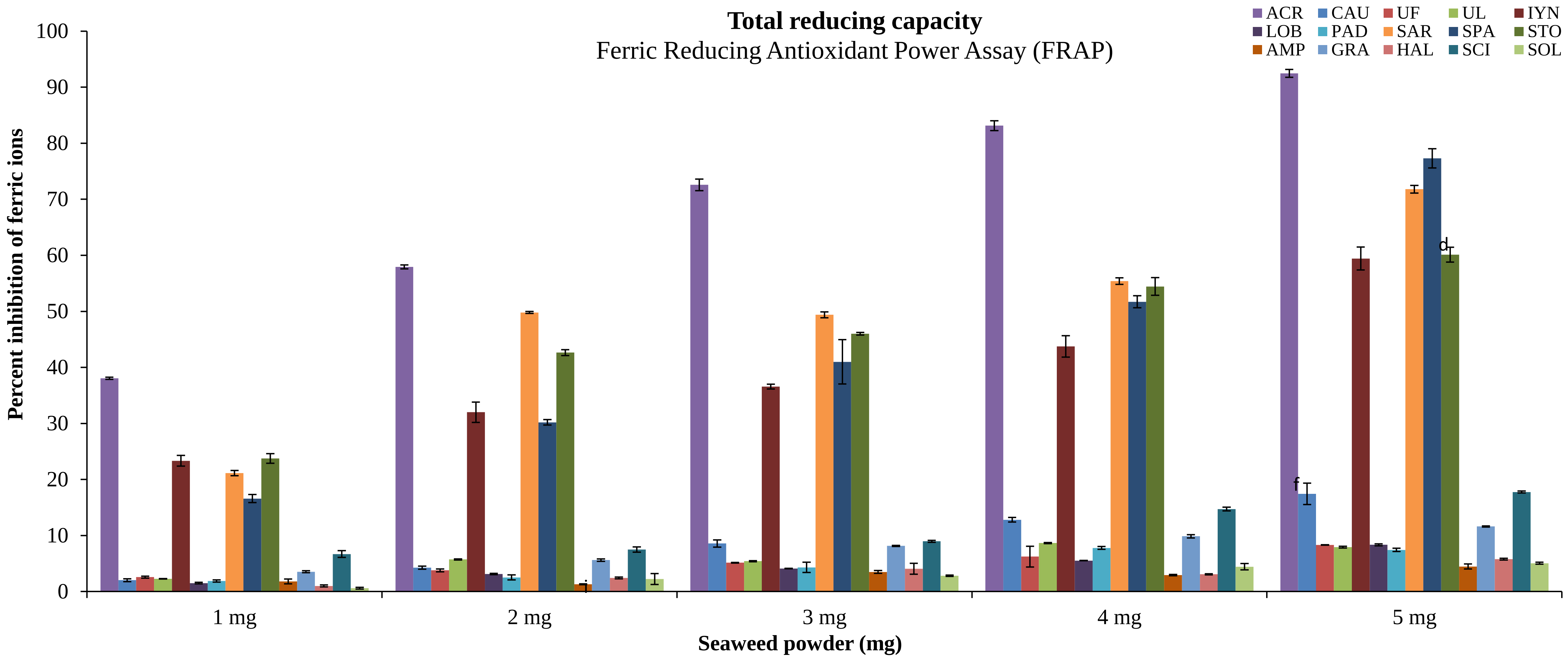

Figure S3: Reducing activity of abundant tropical seaweeds. Activity is measured as percent inhibition of ferric ions free radicals, expressed as mean ± standard error of the mean (SE; n=3). ACR: *Acrosiphonia orientalis*; CAU: *Caulerpa scalpelliformis*; UF: *Ulva fasciata*; UL: *Ulva lactuca;* IYN: *Iyengaria stellata*; LOB: *Lobophora variegata*; PAD: *Padina boergesenii*; SAR: *Sargassum linearifolium*; SPA: *Spatoglossum asperum*; STO: *Stoechospermum marginatum*; AMP: *Amphiroa anceps*; GRA: *Grateloupia indica*; HAL: *Halymenia porphyriformis*; SCI: *Scinaia carnosa*; SOL: *Solieria chordalis*

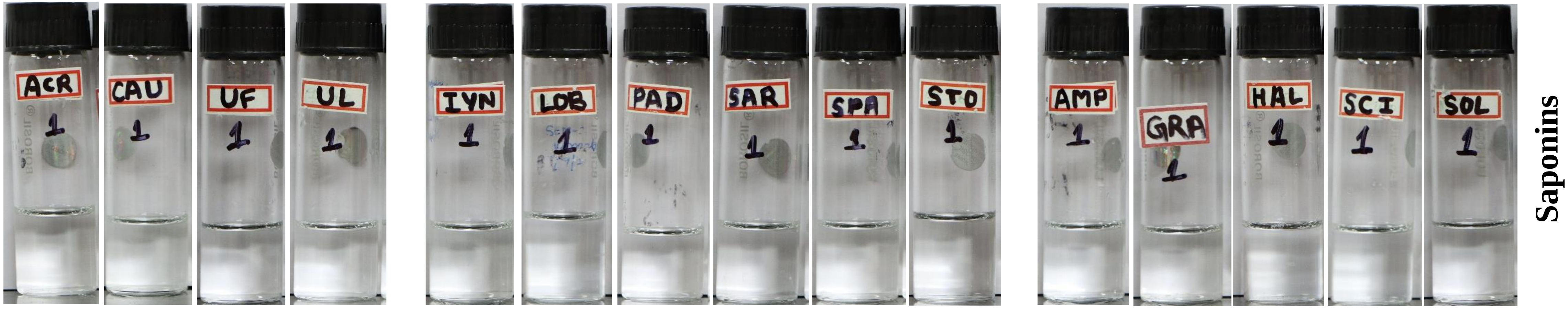

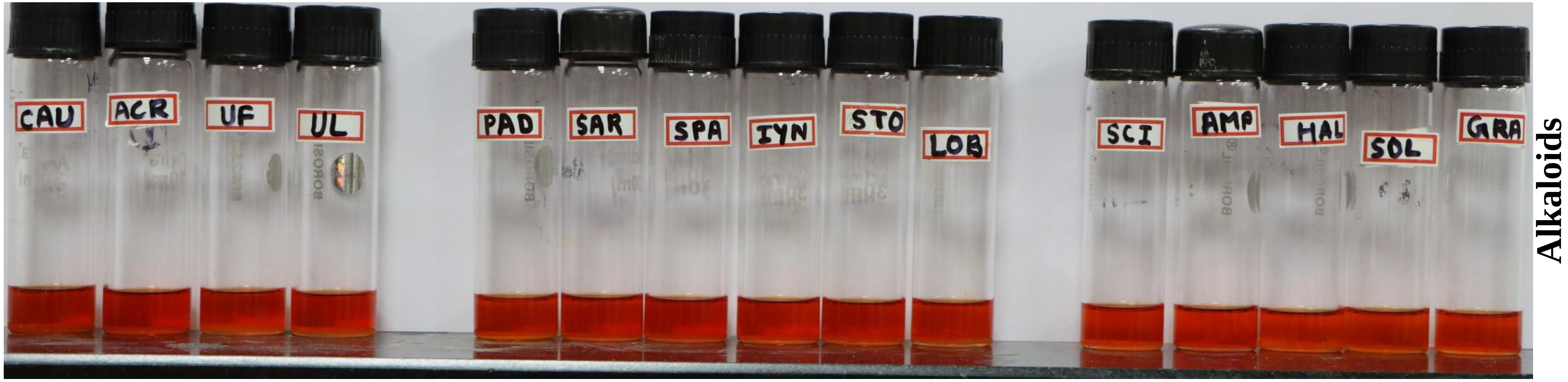

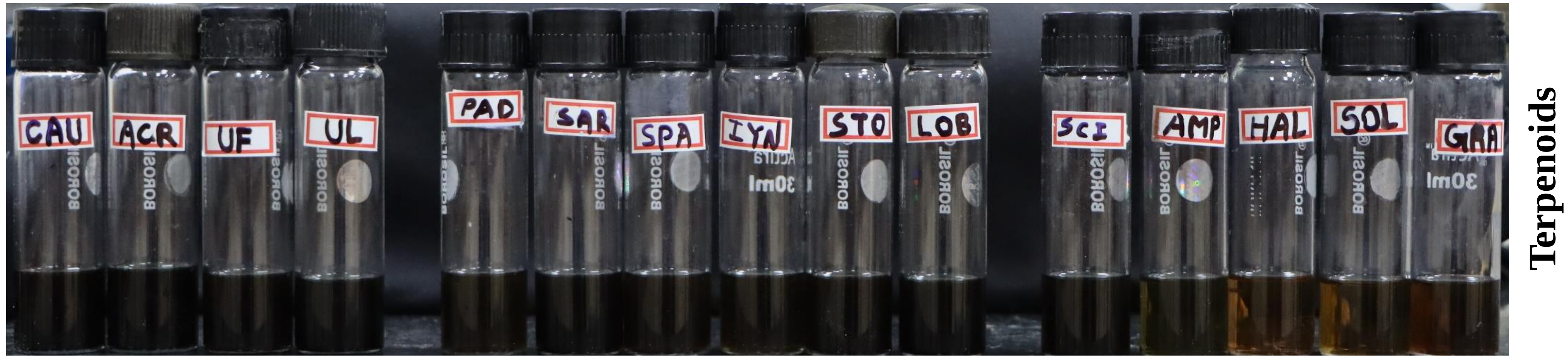

Figure S4: Estimation of the presence of anti-nutritional compounds in abundant tropical seaweeds. Anti-nutritional compounds including saponins, alkaloids and terpenoids were determined in abundant tropical seaweeds.

ACR: *Acrosiphonia orientalis*; CAU: *Caulerpa scalpelliformis*; UF: *Ulva fasciata*; UL: *Ulva lactuca;* IYN: *Iyengaria stellata*; LOB: *Lobophora variegata*; PAD: *Padina boergesenii*; SAR: *Sargassum linearifolium*; SPA: *Spatoglossum asperum*; STO: *Stoechospermum marginatum*; AMP: *Amphiroa anceps*; GRA: *Grateloupia indica*; HAL: *Halymenia porphyriformis*; SCI: *Scinaia carnosa*; SOL: *Solieria chordalis*
